# Genome sequence of the H_2_-producing *Clostridium beijerinckii* strain Br21 isolated from a sugarcane vinasse treatment plant

**DOI:** 10.1590/1678-4685-GMB-2017-0315

**Published:** 2019-01-31

**Authors:** Bruna Constante Fonseca, Diego Mauricio Riaño-Pachón, María-Eugenia Guazzaroni, Valeria Reginatto

**Affiliations:** 1 Universidade de São Paulo Universidade de São Paulo Faculdade de Filosofia Ciências e Letras de Ribeirão Preto Departamento de Química Ribeirão PretoSP Brazil Laboratório de Biotecnologia Ambiental e Energias Renováveis (LABIORE), Departamento de Química, Faculdade de Filosofia Ciências e Letras de Ribeirão Preto, Universidade de São Paulo, Ribeirão Preto, SP, Brazil; 2 Universidade de São Paulo Universidade de São Paulo Instituto de Química Departamento de Bioquímica São PauloSP Brazil Laboratório de Biologia de Sistemas Regulatórios, Departamento de Bioquímica, Instituto de Química, Universidade de São Paulo, São Paulo, SP, Brazil; 3 Universidade de São Paulo Universidade de São Paulo Faculdade de Filosofia Ciências e Letras de Ribeirão Preto Departamento de Biologia Ribeirão PretoSP Brazil Departamento de Biologia, Faculdade de Filosofia Ciências e Letras de Ribeirão Preto, Universidade de São Paulo, Ribeirão Preto, SP, Brazil

**Keywords:** Clostridium, biofuels, biohydrogen, beta-glucosidase

## Abstract

We report on the nearly complete genome sequence of *Clostridium beijerinckii* strain Br21, formerly isolated from a sugarcarne vinasse wastewater treatment plant. The resulting genome is ca. 5.9 Mbp in length and resembles the size of previously published *C. beijerinckii* genomes. We annotated the genome sequence and predicted a total of 5323 genes. Strain Br21 has a genetic toolkit that allows it to exploit diverse sugars that are often found after lignocellulosic biomass pretreatment to yield products of commercial interest. Besides the whole set of genes encoding for enzymes underlying hydrogen production, the genome of the new strain includes genes that enable carbon sources conversion into butanol, ethanol, acetic acid, butyric acid, and the chemical block 1,3-propanediol, which is used to obtain polymers. Moreover, the genome of strain Br21 has a higher number of ORFs with predicted beta-glucosidase activity as compared to other *C. beijerinckii* strains described in the KEGG database. These characteristics make *C. beijerinckii* strain Br21 a remarkable candidate for direct use in biotechnological processes and attest that it is a potential biocatalyst supplier.

Hydrogen (H_2_) has attracted attention because it is an energy carrier with higher energy per unit of weight (120 kJ/g) than fossil fuels, such as petroleum (42 kJ/g) and coal (24 kJ/g). In addition, H_2_ combustion does not emit CO_2_. Besides physical chemical approaches, H_2_ can be obtained by fermentation of renewable materials, such as pure carbohydrates or carbohydrate-rich wastes and wastewater, at low pressure and temperature (Elsharnouby *et al.*, 2013).

Bacteria in the genus *Clostridium*, mainly *C. acetobutylicum* and *C. beijerinckii,* can generate various products of industrial interest, including H_2_ (Elsharnouby *et al.*, 2013). During their exponential growth phase, these bacteria excrete acetate, butyrate, H_2_, and CO_2_ (Schiel-Bengelsdorf *et al.*, 2013). At the end of the exponential growth phase, these bacteria take up acetate and butyrate and convert them into acetone, butanol, and ethanol in the so-called ABE fermentation (solventogenesis), and start endospore synthesis (Jones and Woods, 1986; Schiel-Bengelsdorf *et al.*, 2013). Elucidating the acidogenesis, solventogenesis, and sporulation metabolic networks is crucial if we are to take advantage of this metabolism to obtain desired industrial products.

We present the genome sequence of a new isolate within the phylum Firmicutes, namely the bacterial strain Br21 belonging to the family Clostridiaceae. This bacteria was previously isolated from a sludge collected from an Upflow Anaerobic Sludge Blanked (UASB) bioreactor employed to treat wastewater from a sugar and ethanol production plant. To ensure the emergence of spore-forming bacteria, we acidified the sludge at pH 3.0 for 12 h before isolating the new *Clostridium* strain, as described previously (Fonseca *et al.*, 2016).

The ability of the new isolate to produce H_2_ from different monosaccharides was assayed in a preceding work. Strain Br21 affords the highest H_2_ yield using glucose, galactose, mannose, and xylose, that are the main biomass substrates (Fonseca *et al.*, 2016). In the same formerly work, Strain Br21 16S rRNA gene was sequenced (GenBank accession no. KT626859) revealing that this strain is affiliated to the family Clostridiaceae (order Clostridiales) and has 99.78% 16S rRNA gene sequence identity with *C. beijerinckii* NCIMB 8052 and *C. diolis* DSM 5431 as the two most closely related, validly described species (Fonseca *et al.*, 2016). However, to confirm the new isolate identity, as well as to get deeper insight about its biotechnological potential its whole genome was sequenced as described below.

Bacterial cells were imaged by high-resolution scanning electron microscopy (SEM) (JEOL, Ltd.; Tokyo, Japan) (Figure S1). After 24 h, the cells consisted of elongated and round straight bacterial stems measuring ca. 3-8 μm × 0.7 μm (Figure S1 A,B). At the end of the exponential growth phase (at 60 h), stem-shaped cells began to form endospores (Figure S1 C). All the morphological characteristics described above agree with literature data for *C. beijerinckii* (Jones and Woods, 1986).

For genome sequencing, we obtained strain Br21 DNA from a cell pellet after cultivating the bacterium in liquid CH medium for 48 h, as described in Fonseca *et al.* (2016). We generated one short insert size paired-end library by using the Nextera DNA preparation kit and an additional long-insert library, 5-7 Kbp, with the Nextera Mate Pair Library preparation kit. We sequenced both libraries on HiSeq2500, which produced a total of ~1.4 x 10^7^ reads (2 x 100 bp). We preprocessed mate pair and paired-end reads with FastQC (http://www.bioinformatics.babraham.ac.uk/projects/fastqc/) and Trimmomatic (Bolger *et al.*, 2014). Mate-pair reads were further processed with NExtClip and types A, B, and C reads were kept for *de novo* assembly and scaffolding (Leggett *et al.*, 2014). We estimated genome size by kmer statistics with Kmergenie (Chikhi and Medvedev, 2014). The high-quality reads were assembled in SPAdes v3.9.0 (Bankevich *et al.*, 2012). The resulting genome assembly is ~5.9 Mbp in length (99.8% of the predicted genome size), similar in size to previously published *C. beijerinckii* genomes (https://goo.gl/3SgkeS), with a final coverage of 230x.

This Whole Genome Shotgun project was deposited at DDBJ/ENA/GenBank under the accession number MWMH00000000. The version described in this paper is version MWMH01000000.

The assembly has 28 scaffolds; the longest is 1.19 Mbp with mean, median, and N50 lengths of 214,033.75 bp, 81,771 bp, and 604,572 bp, respectively. The genome assembly was annotated with the NCBI Prokaryotic Genome Annotation Pipeline (Tatusova *et al.*, 2016), which predicted a total of 5323 genes, of which 5099, 16, 54, 7, 147, and 1 encode for proteins, rRNA genes, tRNAs, ncRNAs, putative pseudogenes, and CRISPR array, respectively. The 16S rDNA phylogenetic tree shown in Figure 1 (Table S1) places strain Br21 in a clade with *C. beijerincki* and *C. diolis*, although with low bootstrap support, which prompted us to carry out genome-wide analyses to confirm strain assignment to the species level. The multilocus phylogenetic tree shown in Figure S2 (Table S2) was inferred based on a set o 168 single-copy gene markers, proposed to resolve phylogenetic relationships among Firmicutes (Wang and Wu, 2013). The multilocus tree clearly shows all *C. beijerincki* strains, as well as the single *C. diolis* strain and strain Br21 forming a clade with 100% bootstrap support. Particularly, Br21 forms a subclade (with 100% bootstrap support) with the *C. beijerincki* strains DSM 53, NRRL B-593 and NRRL B-528 (individual gene alignments and phylogenetic trees are available under DOI:10.6084/m9.figshare.5993164). Further genome-wide comparisons conducted with the Genome-to-Genome distance calculator (Meier-Kolthoff *et al.*, 2013) revealed that strain Br21 has a predicted DNA-DNA hybridization value (DDH) of 66.3%, 65.3%, and 76.5% against *C. diolis* DSM 15410, *C. beijerinckii* NCIMB 8052, and *C. beijerinckii* NRRL B-528, respectively (Table S3). DDH values above 70% are required for species-level assignment. Computation of genome wide ANI values and alignment fraction are being increasingly used in bacterial taxonomy and have been posited as objective and precise criteria for bacterial species delimitation (Varghese *et al.*, 2015). The OrthoANIu (Lee *et al.*, 2016) values are 96.18%, 96.12%, and 97.61% respectively, with 95%-96% being usually used as cut-off for species demarcation (Figure 2, Table S3). The DDH of *C. diolis* DSM 15410 *vs C. beijerinckii* NCIMB 8052 is 79.2%, their OrthoANIu is 97.74%, suggesting that they are members of the same species, in agreement with recent findings (Figure 1 and Figure S1; Poehlein *et al.*, 2017). An extended description of the procedures followed to assign strain Br21 to the species level is available in Supplementary Material Text S1.

**Figure 1:**
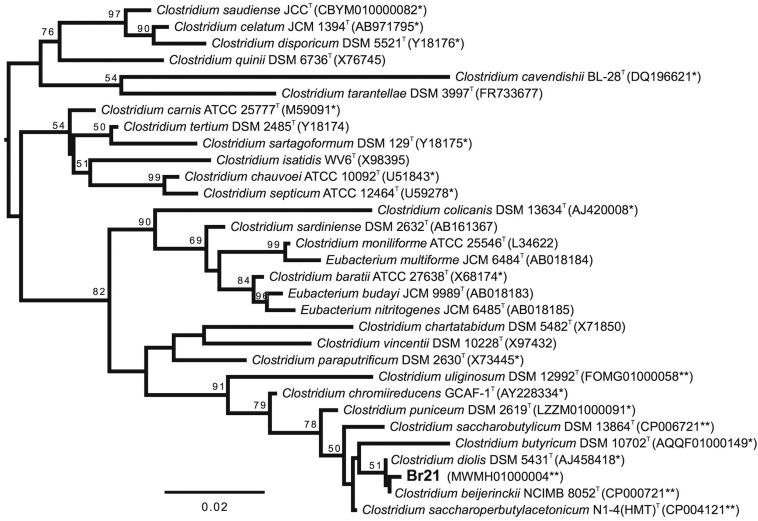
Maximum likelihood phylogenetic tree based on the 16S rDNA sequences, representing the evolutionary relationships between strain Br21 (in bold) and closely related strains in the genus *Clostridium*. The scale shows 0.02 nucleotide changes per nucleotide position. Sequences with at least 94% identity to the 16S rDNA sequence extracted from the strain Br21 genome sequence were identified with the EzBiocloud identification tool and kept for further analysis. Sequences were aligned with MAFFT’s Q-INS-i option. Phylogeny was inferred with RAxML under the GTR+Γ+I evolutionary model, with automatic bootstrapping. (T) Type strain. (*) At least one strain in the species has had its genome sequenced. (**) The 16S rDNA sequence was extracted from the genome sequence. See Text S1 for further details and Table S1 for details of the sequences included.

**Figure 2:**
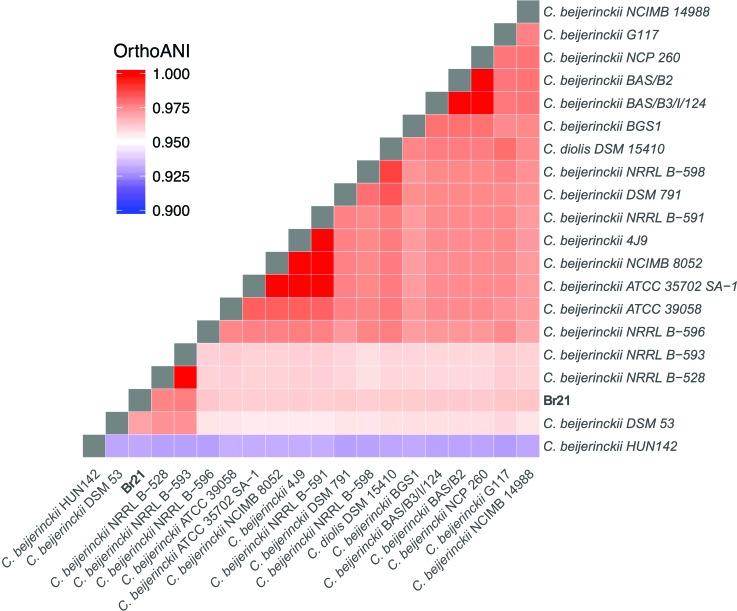
Heatmap of OrthoANIu results among members of *C. beijerincki*, *C. diolis* and Br21. Values above 95%, usually considered as species boundary, appear in shades of red. Strain HUN142 is very distinct from any other *C. beijerincki* strain. Strain DSM 15419 formally assigned to *C. diolis*, cannot be distinguished from the main group of *C. beijerincki* strains. Strain Br21 forms a subcluster (lower left corner) together with *C. beijerincki* strains DSM 53, NRRL B-593 and NRRL B-528, a group that is also supported by the multilocus phylogenetic analysis (Figure S2). See Text S1 for further details and Table S1 for details of the sequences included.

According to Biebl and Spröer (2002), some *C. diolis* strains are very close to *C. beijerinckii* (as judged from molecular analyses) and not very distant in terms of the DNA-DNA hybridization data, but growth and nutrition differences suggested their classification as a separate species. For example, unlike *C. beijerinckii*, *C. diolis* does not ferment starch, raffinose, or inositol (Biebl and Spröer, 2002). Experiments conducted in our laboratory showed that strain Br21 can grow by consuming starch, raffinose, or inositol as the only carbon source, giving H_2_ as product (data not shown). Thus, we named strain Br21 as *C. beijerinckii*. The phylogenetic analyses, the predicted DDH values and the OrthoANIu results support strain Br21 classification as *C. beijerinckii*, being strains NRRL B-593, NRRL B-528, and DSM 53 its closest relatives, within a cohesive and distinct clade.

We divided the enzymes identified in the *C. beijerinckii* Br21 genome into functional classes according to the EC nomenclature so that we could focus on the substrate specificity range of the enzyme catalogue, with especial emphasis on the glycosyl hydrolase group (EC 3.2.1.-). We detected 49 genes encoding for enzymes that hydrolyze or modify sugars, including α-galactose, cellobiose, starch, glycogen, maltose, chitin, pullulan, 6-phospho-D-glucosides (including 6-phospho-beta-D-glucosyl-(1,4)-D-glucose, trehalose-6-phosphate, and sucrose 6-phosphate), xylose, alpha-D-mannose, and α-L-arabinosides (Figure 3, Table S4). Accordingly, former experimental data showed strain Br21 can grow and produce H_2_ from glucose, sucrose, xylose, cellobiose, and starch (Fonseca *et al.*, 2016). Closely related bacteria like *C. beijerinckii* NCIMB 8052 and ATCC 35702 present a slightly higher number of total genes encoding for glycosyl hydrolases (58 and 61, respectively) as compared to the 49 genes identified in strain Br21. Unexpectedly, strain NCIMB 8052 does not contain genes encoding for alpha-mannosidases (EC 3.2.1.24), alpha-xylosidases (EC 3.2.1.-), or neopullulanases (EC 3.2.1.135), and strain ATCC 35702 does not have genes for alpha-mannosidases (EC 3.2.1.24) and neopullulanases (EC 3.2.1.135) in their genomes, as revealed by searches in the KEGG database (Kanehisa and Goto, 2000).

**Figure 3:**
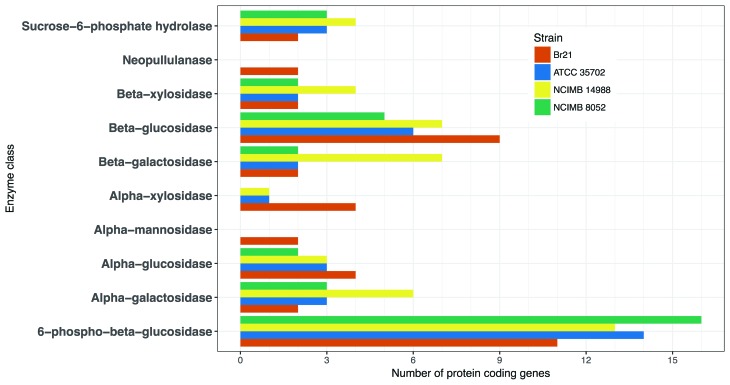
Distribution of glycosyl hydrolases identified in the genome of *C. beijerinckii* Br21 and related strains (NCIMB 8052, ATCC 35702 and NCIMB 14988) based on their function as defined by the fourth level of EC nomenclature. Only enzymes for which the relative percentage is higher than 5% relative to the total are specifically shown.

Interestingly, strain Br21 has a larger number of ORFs with predicted beta-glucosidase activity (EC 3.2.1.21; 9 genes) as compared to *C. beijerinckii* NCIMB 8052 and ATCC 35702 (5 and 6 ORFs, respectively) (Figure 3, Table S4). Beta-glucosidases (beta-D-glucoside glucohydrolases, EC 3.2.1.21) have a key role in cellulose hydrolysis, as they complete the final degradation step (Singhania *et al.*, 2013). These enzymes have recently attracted attention due to their functions in the production of bioethanol and other biofuels from agricultural residues (Singhania *et al.*, 2013).

As mentioned above, the *C. beijerinckii* Br21 genome comprises 49 genes encoding glycosyl hydrolases; some of these genes are absent in the genomes of closely related strains (Figure 3, Table S4). This corresponds to the hydrolysis of (1,6)-α-, (1,2)-α-, (1,4)-β-, (1,4)-α-branch linkages, which is known to play essential roles in the sugar industry, pulp and paper industry, as well as in medicine (Ferrer *et al.*, 2016).

Concerning *C. beijerinckii* Br21 application in biofuel production, the genome analysis showed the presence of genes encoding for the electron carriers and enzymes involved in H_2_ evolution and butanol fermentation. *C. beijerinckii* Br21 has 19, 3, and 5 genes, respectively, encoding for the electron carrier ferredoxin, pyruvate-flavodoxin oxidoreductase-PFOR (EC 1.2.7.-), and Fe-Fe hydrogenase (EC 1.12.7.2), which directly account for H_2_ generation (Table S5). Moreover, the Br21 strain genome presents the most important genes encoding for the enzymes underlying butanol production, including genes related to acetyl-CoA acetyltransferase or -thiolase (EC 2.3.1.9), butyrate-acetoacetate CoA-transferase and acetyl-CoA acetyltransferase (CoA transferase, EC: 2.8.3.9), 3-hydroxybutyryl-CoA dehydrogenase (EC 1.1.1.157), butyryl-CoA dehydrogenase (EC: 1.3.8.1), NADH-dependent butanol dehydrogenase A (EC: 1.1.1.-), and an alcohol dehydrogenase that can transform butyraldehyde into butanol (see Table S5). However, strain Br21 does not present the gene *adc* encoding for acetoacetate decarboxylase, which catalyses the conversion of acetoacetate to acetone and carbon dioxide (Table S5).

Remarkably, *C. beijerinckii* Br21 presents genes encoding for enzymes that convert glycerol into 1,3-propanediol, a high-value chemical block used to produce a thermoplastic for the textile and automobile industries (Papanikolaou *et al.*, 2000). The gene encoding for enzyme 1,3-propanediol dehydrogenase (EC 1.1.1.202) appears in its genome (Table S5), but not in the *C. beijerinckii* NCIMB 8052 and ATCC 35702 genomes, as revealed by KEGG database analysis. 1,3-Propanediol generation by *C. beijerinckii* DSM 791 has been described only recently (Wischral *et al.*, 2016).

Strain Br21 genome analysis highlights its biotechnological potential, particularly regarding its use in the production of biofuels and chemicals from a wide spectrum of substrates.

## Supplementary material

The following online material is available for this article:

Text S1 Extended description of the procedures followed for species level assignment of strain Br21.

Table S1 Details about the strains shown in Figure 1.

Table S2 Details about the genomes used for strain identification using genome-wide information.

Table S3 Complete results from the Genome-to-Genome Distance Calculator and OrthoANIu.

Table S4 Genes encoding for glycosyl hydrolases identified in the *C. beijerinckii* strain Br21 genome.

Table S5 Genes encoding for enzymes and electron carriers related to biofuel and chemical production identified in the *C. beijerinckii* strain Br21 genome.

Figure S1 Scanning electron micrograph of *C. beijerinckii* strain Br21 during the logarithmic growth phase.

Figure S2 Maximum-likelihood phylogeny with 168 markers for the genome sequences of all clostridia deposited in the NCBI RefSeq database
