# Supplementary material for: Genome sequence of the H2-producing Clostridium beijerinckii strain Br21 isolated from a sugarcane vinasse treatment plant
Source: Genet Mol Biol. 2019 Jan 31;42(1):139–44. doi: 10.1590/1678-4685-GMB-2017-0315 (PMC6428130; doi:10.1590/1678-4685-GMB-2017-0315)

Supplementary Material "Genome sequence of the H_2-producing *Clostridium beijerinckii* strain Br21 isolated from a sugarcane vinasse treatment plant"

Figure S1- Scanning electron micrograph of *C. beijerinckii* strain Br21 during the logarithmic growth phase, at 24 h (A and B), and at 60 h (C) in the stationary growth phase with the beginning of endospore formation.

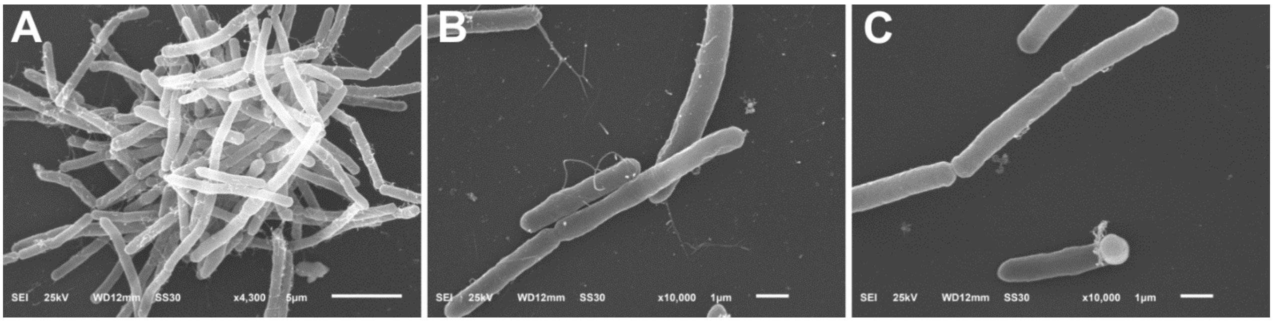

Supplement: Supplementary file 1 [file 1415-4757-GMB-1678-4685-GMB-2017-0315-s007.pdf]
