# Supplementary material for: Genome sequence of the H2-producing Clostridium beijerinckii strain Br21 isolated from a sugarcane vinasse treatment plant
Source: Genet Mol Biol. 2019 Jan 31;42(1):139–44. doi: 10.1590/1678-4685-GMB-2017-0315 (PMC6428130; doi:10.1590/1678-4685-GMB-2017-0315)
Supplement: Supplementary file 2 [file 1415-4757-GMB-1678-4685-GMB-2017-0315-s002.pdf]

Supplementary Material "Genome sequence of the H_2-producing *Clostridium beijerinckii* strain Br21 isolated from a sugarcane vinasse treatment plant"

Table S1 - Details about the strains shown in Figure 1

Rank	Name	Strain	Accession	Origin of Sequence	Pairwise Similarity(%)	Mismatch / Total nt	Completeness(%)
**0**	***Clostridium* sp. Br21**	**Br21**	**MWMH01000004**	**Genome**	**-**	**-**	**97.1**
1	*Clostridium beijerinckii*	NCIMB 8052	CP000721	Genome	99.8	3/1392	100
2	*Clostridium diolis*	DSM 5431	AJ458418	Single	99.8	3/1391	100
3	*Clostridium saccharoperbutylacetonicum*	N1-4(HMT)	CP004121	Genome	99	14/1392	100
4	*Clostridium chromiireducens*	GCAF-1	AY228334	Single	99	14/1391	100
5	*Clostridium puniceum*	DSM 2619	LZZM01000091	Single	98.9	16/1392	100
6	*Clostridium saccharobutylicum*	DSM 13864	CP006721	Genome	98.3	23/1392	100
7	*Clostridium butyricum*	DSM 10702	AQQF01000149	Single	97.8	30/1392	100
8	*Clostridium paraputrificum*	DSM 2630	X73445	Single	96.7	46/1391	99.9
9	*Clostridium uliginosum*	DSM 12992	FOMG01000058	Genome	96.5	49/1392	100
10	*Clostridium chartatabidum*	DSM 5482	X71850	Single	96.3	51/1389	100
11	*Clostridium sardiniense*	DSM 2632	AB161367	Single	96.1	54/1391	100
12	*Clostridium saudiense*	JCC	CBYM010000082	Single	95.8	58/1392	100
13	*Clostridium baratii*	ATCC 27638	X68174	Single	95.8	58/1391	100
14	*Clostridium tertium*	DSM 2485	Y18174	Single	95.8	59/1392	100

Rank	Name	Strain	Accession	Origin of Sequence	Pairwise Similarity(%)	Mismatch / Total nt	Completeness(%)
15	*Clostridium vincentii*	DSM 10228	X97432	Single	95.6	61/1392	100
16	*Clostridium sartagoforme*	DSM 1292	Y18175	Single	95.6	61/1392	100
17	*Clostridium disporicum*	DSM 5521	Y18176	Single	95.4	64/1392	100
18	*Clostridium celatum*	JCM 1394	AB971795	Single	95.4	64/1392	100
19	*Clostridium carnis*	ATCC 25777	M59091	Single	95.4	63/1369	99.4
20	*Eubacterium budayi*	JCM 9989	AB018183	Single	95.4	64/1388	100
21	*Clostridium moniliforme*	ATCC 25546	L34622	Single	95.4	63/1361	99.9
22	*Clostridium colicanis*	DSM 13634	AJ420008	Single	95.3	65/1392	99.9
23	*Eubacterium multiforme*	JCM 6484	AB018184	Single	95.3	65/1386	100
24	*Eubacterium nitritogenes*	JCM 6485	AB018185	Single	95.2	66/1388	100
25	*Clostridium quinii*	DSM 6736	X76745	Single	95.1	68/1388	99.9
26	*Clostridium isatidis*	WV6	X98395	Single	95.1	65/1323	92.7
27	*Clostridium tarantellae*	DSM 3997	FR733677	Single	95	70/1390	100
28	*Clostridium cavendishii*	BL-28	DQ196621	Single	94.8	72/1373	98.8
29	*Clostridium chauvoei*	ATCC 10092	U51843	Single	94.8	73/1392	100
30	*Clostridium septicum*	ATCC 12464	U59278	Single	94.8	73/1391	100
