# Supplementary material for: Genome sequence of the H2-producing Clostridium beijerinckii strain Br21 isolated from a sugarcane vinasse treatment plant
Source: Genet Mol Biol. 2019 Jan 31;42(1):139–44. doi: 10.1590/1678-4685-GMB-2017-0315 (PMC6428130; doi:10.1590/1678-4685-GMB-2017-0315)
Supplement: Supplementary file 3 [file 1415-4757-GMB-1678-4685-GMB-2017-0315-s008.pdf]

Supplementary Material "Genome sequence of the H_2-producing *Clostridium beijerinckii* strain Br21 isolated from a sugarcane vinasse treatment plant"

Figure S2. Maximum-likelihood phylogeny with 168 markers. The genome sequences of all clostridia deposited in NCBI's RefSeq were employed. The sequences of the 168 phylogenetic markers for Firmicutes were identified with Phyla_AMPHORA (http://wolbachia.biology.virginia.edu/WuLab/Software.html) with the script "MarkerScanner.pl",and aligned and trimmed with the script "MarkerAlignTrim.pl". Proper evolutionary model for each marker was determined with ProtTest v3.4.2. Phylogenetic inference was performed with RAxML v8.2.10 by using partitions (one partition for each marker with their own evolutionary model) and rapid bootstrapping with 200 replicates, which were automatically determined with the autoMR criterion. Only bootstrap values equal to or higher than 50% are shown. The RefSeq genome access number appears to the left of the species name. All *C. beijerinckii* strains form a monophyletic clade with 100% bootstrap support; this clade includes the single available *C. diolis* strain DSM 15410. The closest strains to Br21 are NRRL B−593, NRRL B−528, and DSM 53.

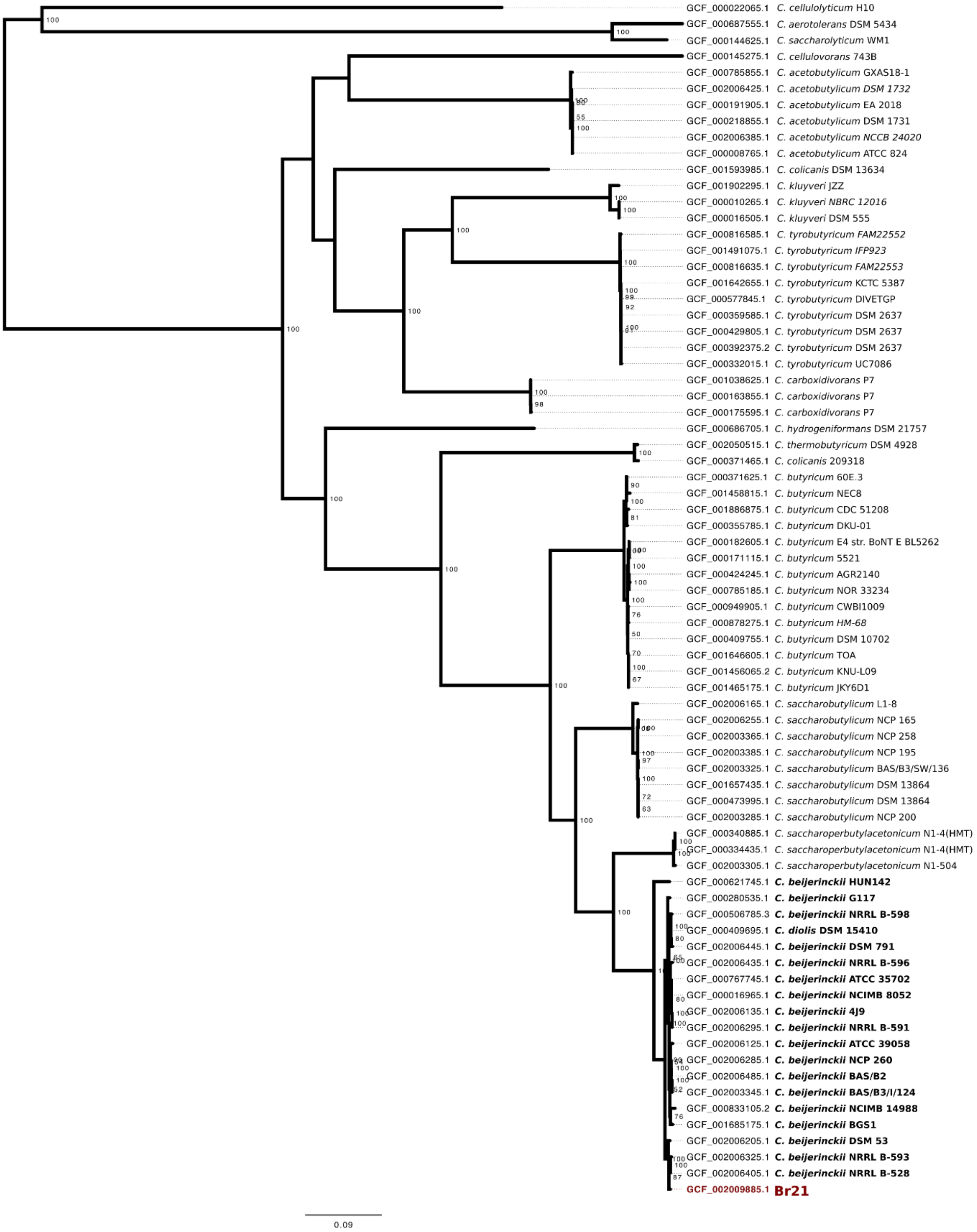

GCF_000022065.1 C. cellulolyticum H10
GCF_000687555.1 C. aerotolerans DSM 5434
GCF_000144625.1 C. saccharolyticum WM1
GCF_000145275.1 C. cellulovorans 743B
GCF_000785855.1 C. acetobutylicum GXAS18-1
GCF_002006425.1 C. acetobutylicum DSM 1732
GCF_000191905.1 C. acetobutylicum EA 2018
GCF_000218855.1 C. acetobutylicum DSM 1731
GCF_002006385.1 C. acetobutylicum NCCB 24020
GCF_000008765.1 C. acetobutylicum ATCC 824
GCF_001593985.1 C. colicanis DSM 13634
GCF_001902295.1 C. kluyveri JZZ
GCF_000010265.1 C. kluyveri NBRC 12016
GCF_000016505.1 C. kluyveri DSM 555
GCF_000816585.1 C. tyrobutyricum FAM22552
GCF_001491075.1 C. tyrobutyricum IFP923
GCF_000816635.1 C. tyrobutyricum FAM22553
GCF_001642655.1 C. tyrobutyricum KCTC 5387
GCF_000577845.1 C. tyrobutyricum DIVETGP
GCF_000359585.1 C. tyrobutyricum DSM 2637
GCF_000429805.1 C. tyrobutyricum DSM 2637
GCF_000392375.2 C. tyrobutyricum DSM 2637
GCF_000332015.1 C. tyrobutyricum UC7086
GCF_001038625.1 C. carboxidivorans P7
GCF_000163855.1 C. carboxidivorans P7
GCF_000175595.1 C. carboxidivorans P7
GCF_000686705.1 C. hydrogeniformans DSM 21757
GCF_002050515.1 C. thermobutyricum DSM 4928
GCF_000371465.1 C. colicanis 209318
GCF_000371625.1 C. butyricum 60E.3
GCF_001458815.1 C. butyricum NEC8
GCF_001886875.1 C. butyricum CDC 51208
GCF_000355785.1 C. butyricum DKU-01
GCF_000182605.1 C. butyricum E4 str. BoNT E BL5262
GCF_000171115.1 C. butyricum 5521
GCF_000424245.1 C. butyricum AGR2140
GCF_000785185.1 C. butyricum NOR 33234
GCF_000949905.1 C. butyricum CWBI1009
GCF_000878275.1 C. butyricum HM-68
GCF_000409755.1 C. butyricum DSM 10702
GCF_001646605.1 C. butyricum TOA
GCF_001456065.2 C. butyricum KNU-L09
GCF_001465175.1 C. butyricum JKY6D1
GCF_002006165.1 C. saccharobutylicum L1-8
GCF_002006255.1 C. saccharobutylicum NCP 165
GCF_002003365.1 C. saccharobutylicum NCP 258
GCF_002003385.1 C. saccharobutylicum NCP 195
GCF_002003325.1 C. saccharobutylicum BAS/B3/SW/136
GCF_001657435.1 C. saccharobutylicum DSM 13864
GCF_000473995.1 C. saccharobutylicum DSM 13864
GCF_002003285.1 C. saccharobutylicum NCP 200
GCF_000340885.1 C. saccharoperbutylacetonicum N1-4(HMT)
GCF_000334435.1 C. saccharoperbutylacetonicum N1-4(HMT)
GCF_002003305.1 C. saccharoperbutylacetonicum N1-504
GCF_000621745.1 C. beijerinckii HUN142
GCF_000280535.1 C. beijerinckii G117
GCF_000506785.3 C. beijerinckii NRRL B-598
GCF_000409695.1 C. diolis DSM 15410
GCF_002006445.1 C. beijerinckii DSM 791
GCF_002006435.1 C. beijerinckii NRRL B-596
GCF_000767745.1 C. beijerinckii ATCC 35702
GCF_000016965.1 C. beijerinckii NCIMB 8052
GCF_002006135.1 C. beijerinckii 4J9
GCF_002006295.1 C. beijerinckii NRRL B-591
GCF_002006125.1 C. beijerinckii ATCC 39058
GCF_002006285.1 C. beijerinckii NCP 260
GCF_002006485.1 C. beijerinckii BAS/B2
GCF_002003345.1 C. beijerinckii BAS/B3/I/124
GCF_000833105.2 C. beijerinckii NCIMB 14988
GCF_001685175.1 C. beijerinckii BGS1
GCF_002006205.1 C. beijerinckii DSM 53
GCF_002006325.1 C. beijerinckii NRRL B-593
GCF_002006405.1 C. beijerinckii NRRL B-528
GCF_002009885.1 Br21
0.09
