# Supplementary material for: Genome sequence of the H2-producing Clostridium beijerinckii strain Br21 isolated from a sugarcane vinasse treatment plant
Source: Genet Mol Biol. 2019 Jan 31;42(1):139–44. doi: 10.1590/1678-4685-GMB-2017-0315 (PMC6428130; doi:10.1590/1678-4685-GMB-2017-0315)
Supplement: Supplementary file 4 [file 1415-4757-GMB-1678-4685-GMB-2017-0315-s003.pdf]

Supplementary Material “Genome sequence of the H_2-producing *Clostridium beijerinckii* strain Br21 isolated from a sugarcane vinasse treatment plant”

Table S2 - Details about the genomes used for strain identification using genome-wide information. These genomes were used for the analysis of predicted DNA-DNA hybridization values, orthoANIu values (Figure 2) and the multi-locus phylogeny (Figure S2).

Assembly accession	BioProject	BioSample	Organism name	Infraspecific name	Genome status	Release date	Assembly name	FTP path
GCF_000008765.1	PRJNA57677	SAMN02603243	Clostridium acetobutylicum ATCC 824	strain=ATCC 824	Complete Genome	11/22/02	ASM876v1	ftp://ftp.ncbi.nlm.nih.gov/genomes/all/GCF/000/008/765/GCF_000008765.1_ASM876v1
GCF_000191905.1	PRJNA224116	SAMN02603410	Clostridium acetobutylicum EA 2018	strain=EA 2018	Complete Genome	3/8/11	ASM19190v1	ftp://ftp.ncbi.nlm.nih.gov/genomes/all/GCF/000/191/905/GCF_000191905.1_ASM19190v1
GCF_000218855.1	PRJNA224116	SAMN02603396	Clostridium acetobutylicum DSM 1731	strain=DSM 1731	Complete Genome	6/17/11	ASM21885v1	ftp://ftp.ncbi.nlm.nih.gov/genomes/all/GCF/000/218/855/GCF_000218855.1_ASM21885v1
GCF_000785855.1	PRJNA224116	SAMN03112708	Clostridium acetobutylicum	strain=GXAS18-1	Scaffold	11/21/14	ASM78585v1	ftp://ftp.ncbi.nlm.nih.gov/genomes/all/GCF/000/785/855/GCF_000785855.1_ASM78585v1
GCF_002006385.1	PRJNA224116	SAMN05170515	Clostridium acetobutylicum	strain=NCCB 24020	Contig	2/23/17	ASM200638v1	ftp://ftp.ncbi.nlm.nih.gov/genomes/all/GCF/002/006/385/GCF_002006385.1_ASM200638v1
GCF_002006425.1	PRJNA224116	SAMN05170516	Clostridium acetobutylicum	strain=DSM 1732	Contig	2/23/17	ASM200642v1	ftp://ftp.ncbi.nlm.nih.gov/genomes/all/GCF/002/006/425/GCF_002006425.1_ASM200642v1
GCF_000171115.1	PRJNA224116	SAMN02436238	Clostridium butyricum 5521	strain=5521	Contig	4/10/08	ASM17111v1	ftp://ftp.ncbi.nlm.nih.gov/genomes/all/GCF/000/171/115/GCF_000171115.1_ASM17111v1
GCF_000182605.1	PRJNA224116	SAMN02470281	Clostridium butyricum E4 str. BoNT E BL5262	strain=BoNT E BL5262	Scaffold	5/15/09	ASM18260v1	ftp://ftp.ncbi.nlm.nih.gov/genomes/all/GCF/000/182/605/GCF_000182605.1_ASM18260v1
GCF_000355785.1	PRJNA224116	SAMN02469601	Clostridium butyricum DKU-01	strain=DKU-01	Contig	4/5/13	CloButy1.0	ftp://ftp.ncbi.nlm.nih.gov/genomes/all/GCF/000/355/785/GCF_000355785.1_CloButy1.0
GCF_000371625.1	PRJNA224116	SAMN02596761	Clostridium butyricum 60E.3	strain=60E.3	Scaffold	4/19/13	Clos_buty_60E_3_V1	ftp://ftp.ncbi.nlm.nih.gov/genomes/all/GCF/000/371/625/GCF_000371625.1_Clos_buty_60E_3_V1
GCF_000409755.1	PRJNA224116	SAMN02470945	Clostridium butyricum DSM 10702	strain=DSM 10702	Contig	6/10/13	Clostridium butyricum DSM 10702 Genome sequencing	ftp://ftp.ncbi.nlm.nih.gov/genomes/all/GCF/000/409/755/GCF_000409755.1_Clostridium_butyricum_DSM_10702_Genome_sequencing

Assembly accession	BioProject	BioSample	Organism name	Infraspecific name	Genome status	Release date	Assembly name	FTP path
GCF_000424245.1	PRJNA224116	SAMN02441161	Clostridium butyricum AGR2140	strain=AGR2140	Scaffold	7/11/13	ASM42424v1	ftp://ftp.ncbi.nlm.nih.gov/genomes/all/GCF/000/424/245/GCF_000424245.1_ASM42424v1
GCF_000785185.1	PRJNA224116	SAMN03135098	Clostridium butyricum	strain=NOR 33234	Contig	11/18/14	ASM78518v1	ftp://ftp.ncbi.nlm.nih.gov/genomes/all/GCF/000/785/185/GCF_000785185.1_ASM78518v1
GCF_000878275.1	PRJNA224116	SAMN03272540	Clostridium butyricum	strain=HM-68	Contig	2/19/15	ASM87827v1	ftp://ftp.ncbi.nlm.nih.gov/genomes/all/GCF/000/878/275/GCF_000878275.1_ASM87827v1
GCF_000949905.1	PRJNA224116	SAMN02143030	Clostridium butyricum CWBI1009	strain=CWBI1009	Contig	3/10/15	CWBI1009_v1	ftp://ftp.ncbi.nlm.nih.gov/genomes/all/GCF/000/949/905/GCF_000949905.1_CWBI1009_v1
GCF_001456065.2	PRJNA224116	SAMN04293668	Clostridium butyricum	strain=KNU-L09	Complete Genome	12/9/15	ASM145606v2	ftp://ftp.ncbi.nlm.nih.gov/genomes/all/GCF/001/456/065/GCF_001456065.2_ASM145606v2
GCF_001458815.1	PRJNA224116	SAMEA3146292	Clostridium butyricum	strain=NEC8	Contig	2/12/14	CBut	ftp://ftp.ncbi.nlm.nih.gov/genomes/all/GCF/001/458/815/GCF_001458815.1_CBut
GCF_001465175.1	PRJNA224116	SAMN04285352	Clostridium butyricum	strain=JKY6D1	Complete Genome	12/10/15	ASM146517v1	ftp://ftp.ncbi.nlm.nih.gov/genomes/all/GCF/001/465/175/GCF_001465175.1_ASM146517v1
GCF_001646605.1	PRJNA224116	SAMN04527051	Clostridium butyricum	strain=TOA	Complete Genome	5/18/16	ASM164660v1	ftp://ftp.ncbi.nlm.nih.gov/genomes/all/GCF/001/646/605/GCF_001646605.1_ASM164660v1
GCF_001886875.1	PRJNA224116	SAMN04262337	Clostridium butyricum	strain=CDC_51208	Complete Genome	11/28/16	ASM188687v1	ftp://ftp.ncbi.nlm.nih.gov/genomes/all/GCF/001/886/875/GCF_001886875.1_ASM188687v1
GCF_000145275.1	PRJNA224116	SAMN00002614	Clostridium cellulovorans 743B	strain=743B	Complete Genome	8/11/10	ASM14527v1	ftp://ftp.ncbi.nlm.nih.gov/genomes/all/GCF/000/145/275/GCF_000145275.1_ASM14527v1
GCF_000332015.1	PRJNA224116	SAMN02471393	Clostridium tyrobutyricum UC7086	strain=UC7086	Contig	1/17/13	1st assembly	ftp://ftp.ncbi.nlm.nih.gov/genomes/all/GCF/000/332/015/GCF_000332015.1_1st_assembly
GCF_000359585.1	PRJNA224116	SAMN02470572	Clostridium tyrobutyricum DSM 2637 = ATCC 25755 = JCM 11008	strain=ATCC 25755	Contig	4/9/13	ATCC25755	ftp://ftp.ncbi.nlm.nih.gov/genomes/all/GCF/000/359/585/GCF_000359585.1_ATCC25755
GCF_000392375.2	PRJNA224116	SAMN02471386	Clostridium tyrobutyricum DSM 2637 = ATCC 25755 = JCM 11008	strain=DSM 2637	Contig	5/14/13	ASM39237v2	ftp://ftp.ncbi.nlm.nih.gov/genomes/all/GCF/000/392/375/GCF_000392375.2_ASM39237v2
GCF_000429805.1	PRJNA224116	SAMN02440870	Clostridium tyrobutyricum DSM 2637 = ATCC 25755 = JCM 11008	strain=DSM 2637	Scaffold	7/15/13	ASM42980v1	ftp://ftp.ncbi.nlm.nih.gov/genomes/all/GCF/000/429/805/GCF_000429805.1_ASM42980v1
GCF_000577845.1	PRJNA224116	SAMEA3138940	Clostridium tyrobutyricum DIVETGP	strain=DIVETGP	Contig	2/24/14	CTDIVETGP	ftp://ftp.ncbi.nlm.nih.gov/genomes/all/GCF/000/577/845/GCF_000577845.1_CTDIVETGP
GCF_000816585.1	PRJNA224116	SAMN03177790	Clostridium tyrobutyricum	strain=FAM22552	Scaffold	1/8/15	ASM81658v1	ftp://ftp.ncbi.nlm.nih.gov/genomes/all/GCF/000/816/585/GCF_000816585.1_ASM81658v1
GCF_000816635.1	PRJNA224116	SAMN03177791	Clostridium tyrobutyricum	strain=FAM22553	Scaffold	1/8/15	ASM81663v1	ftp://ftp.ncbi.nlm.nih.gov/genomes/all/GCF/000/816/635/GCF_000816635.1_ASM81663v1

Assembly accession	BioProject	BioSample	Organism name	Infraspecific name	Genome status	Release date	Assembly name	FTP path
GCF_001491075.1	PRJNA224116	SAMEA3699157	Clostridium tyrobutyricum	IFP923	Contig	12/15/15	IFP923	ftp://ftp.ncbi.nlm.nih.gov/genomes/all/GCF/001/491/075/GCF_001491075.1_IFP923
GCF_001642655.1	PRJNA224116	SAMN04415495	Clostridium tyrobutyricum	strain=KCTC 5387	Complete Genome	5/12/16	ASM164265v1	ftp://ftp.ncbi.nlm.nih.gov/genomes/all/GCF/001/642/655/GCF_001642655.1_ASM164265v1
GCF_000016965.1	PRJNA224116	SAMN02598276	Clostridium beijerinckii NCIMB 8052	strain=NCIMB 8052	Complete Genome	6/27/07	ASM1696v1	ftp://ftp.ncbi.nlm.nih.gov/genomes/all/GCF/000/016/965/GCF_000016965.1_ASM1696v1
GCF_000280535.1	PRJNA224116	SAMN02470653	Clostridium beijerinckii G117	strain=G117	Scaffold	7/30/12	CloBeiG117, 1.0	ftp://ftp.ncbi.nlm.nih.gov/genomes/all/GCF/000/280/535/GCF_000280535.1_CloBeiG117_1.0
GCF_000506785.3	PRJNA224116	SAMN02427303	Clostridium beijerinckii NRRL B-598	strain=NRRL B-598	Complete Genome	2/17/17	ASM50678v3	ftp://ftp.ncbi.nlm.nih.gov/genomes/all/GCF/000/506/785/GCF_000506785.3_ASM50678v3
GCF_000621745.1	PRJNA224116	SAMN02744012	Clostridium beijerinckii HUN142	strain=HUN142	Scaffold	4/8/14	ASM62174v1	ftp://ftp.ncbi.nlm.nih.gov/genomes/all/GCF/000/621/745/GCF_000621745.1_ASM62174v1
GCF_000767745.1	PRJNA224116	SAMN03120854	Clostridium beijerinckii ATCC 35702	strain=SA-1	Complete Genome	10/22/14	ASM76774v1	ftp://ftp.ncbi.nlm.nih.gov/genomes/all/GCF/000/767/745/GCF_000767745.1_ASM76774v1
GCF_000833105.2	PRJNA224116	SAMN03024437	Clostridium beijerinckii	strain=NCIMB 14988	Complete Genome	2/19/16	ASM83310v2	ftp://ftp.ncbi.nlm.nih.gov/genomes/all/GCF/000/833/105/GCF_000833105.2_ASM83310v2
GCF_001685175.1	PRJNA224116	SAMN05355371	Clostridium beijerinckii	strain=BGS1	Scaffold	7/14/16	ASM168517v1	ftp://ftp.ncbi.nlm.nih.gov/genomes/all/GCF/001/685/175/GCF_001685175.1_ASM168517v1
GCF_002003345.1	PRJNA224116	SAMN05170526	Clostridium beijerinckii	strain=BAS/B3/I/124	Complete Genome	2/17/17	ASM200334v1	ftp://ftp.ncbi.nlm.nih.gov/genomes/all/GCF/002/003/345/GCF_002003345.1_ASM200334v1
GCF_002006125.1	PRJNA224116	SAMN05170528	Clostridium beijerinckii	strain=ATCC 39058	Contig	2/23/17	ASM200612v1	ftp://ftp.ncbi.nlm.nih.gov/genomes/all/GCF/002/006/125/GCF_002006125.1_ASM200612v1
GCF_002006135.1	PRJNA224116	SAMN05170529	Clostridium beijerinckii	strain=4J9	Contig	2/23/17	ASM200613v1	ftp://ftp.ncbi.nlm.nih.gov/genomes/all/GCF/002/006/135/GCF_002006135.1_ASM200613v1
GCF_002006205.1	PRJNA224116	SAMN05170531	Clostridium beijerinckii	strain=DSM 53	Contig	2/23/17	ASM200620v1	ftp://ftp.ncbi.nlm.nih.gov/genomes/all/GCF/002/006/205/GCF_002006205.1_ASM200620v1
GCF_002006285.1	PRJNA224116	SAMN05170527	Clostridium beijerinckii	strain=NCP 260	Contig	2/23/17	ASM200628v1	ftp://ftp.ncbi.nlm.nih.gov/genomes/all/GCF/002/006/285/GCF_002006285.1_ASM200628v1
GCF_002006295.1	PRJNA224116	SAMN05170530	Clostridium beijerinckii	strain=NRRL B-591	Contig	2/23/17	ASM200629v1	ftp://ftp.ncbi.nlm.nih.gov/genomes/all/GCF/002/006/295/GCF_002006295.1_ASM200629v1
GCF_002006325.1	PRJNA224116	SAMN05170532	Clostridium beijerinckii	strain=NRRL B-593	Contig	2/23/17	ASM200632v1	ftp://ftp.ncbi.nlm.nih.gov/genomes/all/GCF/002/006/325/GCF_002006325.1_ASM200632v1
GCF_002006405.1	PRJNA224116	SAMN05170533	Clostridium beijerinckii	strain=NRRL B-528	Contig	2/23/17	ASM200640v1	ftp://ftp.ncbi.nlm.nih.gov/genomes/all/GCF/002/006/405/GCF_002006405.1_ASM200640v1
GCF_002006435.1	PRJNA224116	SAMN05170523	Clostridium beijerinckii	strain=NRRL B-596	Contig	2/23/17	ASM200643v1	ftp://ftp.ncbi.nlm.nih.gov/genomes/all/GCF/002/006/435/GCF_002006435.1_ASM200643v1
GCF_002006445.1	PRJNA224116	SAMN05170524	Clostridium beijerinckii	strain=DSM 791	Contig	2/23/17	ASM200644v1	ftp://ftp.ncbi.nlm.nih.gov/genomes/all/GCF/002/006/445/GCF_002006445.1_ASM200644v1

Assembly accession	BioProject	BioSample	Organism name	Infraspecific name	Genome status	Release date	Assembly name	FTP path
GCF_002006485.1	PRJNA224116	SAMN05170525	Clostridium beijerinckii	strain=BAS/B2	Contig	2/23/17	ASM200648v1	ftp://ftp.ncbi.nlm.nih.gov/genomes/all/GCF/002/006/485/GCF_002006485.1_ASM200648v1
GCF_002009885.1	PRJNA224116	SAMN06328598	Clostridium beijerinckii	strain=Br21	Scaffold	3/2/17	ASM200988v1	ftp://ftp.ncbi.nlm.nih.gov/genomes/all/GCF/002/009/885/GCF_002009885.1_ASM200988v1
GCF_000022065.1	PRJNA224116	SAMN00623037	[Clostridium] cellulolyticum H10	strain=H10; ATCC 35319	Complete Genome	1/13/09	ASM2206v1	ftp://ftp.ncbi.nlm.nih.gov/genomes/all/GCF/000/022/065/GCF_000022065.1_ASM2206v1
GCF_000010265.1	PRJNA224116	SAMD00060959	Clostridium kluyveri NBRC 12016	strain=NBRC 12016	Complete Genome	1/6/09	ASM1026v1	ftp://ftp.ncbi.nlm.nih.gov/genomes/all/GCF/000/010/265/GCF_000010265.1_ASM1026v1
GCF_000016505.1	PRJNA224116	SAMN02603294	Clostridium kluyveri DSM 555	strain=DSM 555	Complete Genome	7/19/07	ASM1650v1	ftp://ftp.ncbi.nlm.nih.gov/genomes/all/GCF/000/016/505/GCF_000016505.1_ASM1650v1
GCF_001902295.1	PRJNA224116	SAMN06094710	Clostridium kluyveri	strain=JZZ	Complete Genome	12/13/16	ASM190229v1	ftp://ftp.ncbi.nlm.nih.gov/genomes/all/GCF/001/902/295/GCF_001902295.1_ASM190229v1
GCF_002050515.1	PRJNA224116	SAMN04516210	Clostridium thermobutyricum DSM 4928	strain=DSM 4928	Contig	3/21/17	ASM205051v1	ftp://ftp.ncbi.nlm.nih.gov/genomes/all/GCF/002/050/515/GCF_002050515.1_ASM205051v1
GCF_000334435.1	PRJNA224116	SAMN02469458	Clostridium saccharoperbutylacetonicum N1-4(HMT)	strain=ATCC 27021	Contig	1/30/13	CloSac1.0	ftp://ftp.ncbi.nlm.nih.gov/genomes/all/GCF/000/334/435/GCF_000334435.1_CloSac1.0
GCF_000340885.1	PRJNA224116	SAMN02603265	Clostridium saccharoperbutylacetonicum N1-4(HMT)	strain=N1-4(HMT)	Complete Genome	2/19/13	ASM34088v1	ftp://ftp.ncbi.nlm.nih.gov/genomes/all/GCF/000/340/885/GCF_000340885.1_ASM34088v1
GCF_002003305.1	PRJNA224116	SAMN05170535	Clostridium saccharoperbutylacetonicum	strain=N1-504	Complete Genome	2/17/17	ASM200330v1	ftp://ftp.ncbi.nlm.nih.gov/genomes/all/GCF/002/003/305/GCF_002003305.1_ASM200330v1
GCF_000687555.1	PRJNA224116	SAMN02743875	[Clostridium] aerotolerans DSM 5434	strain=DSM 5434	Contig	4/8/14	ASM68755v1	ftp://ftp.ncbi.nlm.nih.gov/genomes/all/GCF/000/687/555/GCF_000687555.1_ASM68755v1
GCF_000144625.1	PRJNA224116	SAMN00017134	[Clostridium] saccharolyticum WM1	strain=WM1	Complete Genome	8/5/10	ASM14462v1	ftp://ftp.ncbi.nlm.nih.gov/genomes/all/GCF/000/144/625/GCF_000144625.1_ASM14462v1
GCF_000473995.1	PRJNA224116	SAMN02603278	Clostridium saccharobutylicum DSM 13864	strain=DSM 13864	Complete Genome	10/18/13	ASM47399v1	ftp://ftp.ncbi.nlm.nih.gov/genomes/all/GCF/000/473/995/GCF_000473995.1_ASM47399v1
GCF_001657435.1	PRJNA224116	SAMN02251455	Clostridium saccharobutylicum DSM 13864	strain=DSM 13864	Contig	6/3/16	ASM165743v1	ftp://ftp.ncbi.nlm.nih.gov/genomes/all/GCF/001/657/435/GCF_001657435.1_ASM165743v1
GCF_002003285.1	PRJNA224116	SAMN05170520	Clostridium saccharobutylicum	strain=NCP 200	Complete Genome	2/17/17	ASM200328v1	ftp://ftp.ncbi.nlm.nih.gov/genomes/all/GCF/002/003/285/GCF_002003285.1_ASM200328v1
GCF_002003325.1	PRJNA224116	SAMN05170519	Clostridium saccharobutylicum	strain=BAS/B3/SW/136	Complete Genome	2/17/17	ASM200332v1	ftp://ftp.ncbi.nlm.nih.gov/genomes/all/GCF/002/003/325/GCF_002003325.1_ASM200332v1
GCF_002003365.1	PRJNA224116	SAMN05170521	Clostridium saccharobutylicum	strain=NCP 258	Complete Genome	2/17/17	ASM200336v1	ftp://ftp.ncbi.nlm.nih.gov/genomes/all/GCF/002/003/365/GCF_002003365.1_ASM200336v1
GCF_002003385.1	PRJNA224116	SAMN05170518	Clostridium saccharobutylicum	strain=NCP 195	Complete Genome	2/17/17	ASM200338v1	ftp://ftp.ncbi.nlm.nih.gov/genomes/all/GCF/002/003/385/GCF_002003385.1_ASM200338v1

Assembly accession	BioProject	BioSample	Organism name	Infraspecific name	Genome status	Release date	Assembly name	FTP path
GCF_002006165.1	PRJNA224116	SAMN05170517	Clostridium saccharobutylicum	strain=L1-8	Contig	2/23/17	ASM200616v1	ftp://ftp.ncbi.nlm.nih.gov/genomes/all/GCF/002/006/165/GCF_002006165.1_ASM200616v1
GCF_002006255.1	PRJNA224116	SAMN05170522	Clostridium saccharobutylicum	strain=NCP 165	Contig	2/23/17	ASM200625v1	ftp://ftp.ncbi.nlm.nih.gov/genomes/all/GCF/002/006/255/GCF_002006255.1_ASM200625v1
GCF_000371465.1	PRJNA224116	SAMN02596770	Clostridium colicanis 209318	strain=209318	Scaffold	4/19/13	Clos_coli_209318_V1	ftp://ftp.ncbi.nlm.nih.gov/genomes/all/GCF/000/371/465/GCF_000371465.1_Clos_coli_209318_V1
GCF_001593985.1	PRJNA224116	SAMN04516095	Clostridium colicanis DSM 13634	strain=DSM 13634	Contig	3/18/16	ASM159398v1	ftp://ftp.ncbi.nlm.nih.gov/genomes/all/GCF/001/593/985/GCF_001593985.1_ASM159398v1
GCF_000163855.1	PRJNA224116	SAMN02469315	Clostridium carboxidivorans P7	strain=P7	Scaffold	5/14/10	ASM16385v1	ftp://ftp.ncbi.nlm.nih.gov/genomes/all/GCF/000/163/855/GCF_000163855.1_ASM16385v1
GCF_000175595.1	PRJNA224116	SAMN02441784	Clostridium carboxidivorans P7	strain=P7	Contig	8/5/09	ASM17559v1	ftp://ftp.ncbi.nlm.nih.gov/genomes/all/GCF/000/175/595/GCF_000175595.1_ASM17559v1
GCF_001038625.1	PRJNA224116	SAMN03740584	Clostridium carboxidivorans P7	strain=P7	Complete Genome	6/24/15	ASM103862v1	ftp://ftp.ncbi.nlm.nih.gov/genomes/all/GCF/001/038/625/GCF_001038625.1_ASM103862v1
GCF_000409695.1	PRJNA224116	SAMN02470960	Clostridium diolis DSM 15410	strain=DSM 15410	Contig	6/10/13	Clostridium diolis DSM 15410 Genome sequencing	ftp://ftp.ncbi.nlm.nih.gov/genomes/all/GCF/000/409/695/GCF_000409695.1_Clostridium_diolis_DSM_15410_Genome_sequencing
GCF_000686705.1	PRJNA224116	SAMN02743370	Clostridium hydrogeniformans DSM 21757	strain=DSM 21757	Scaffold	5/8/14	ASM68670v1	ftp://ftp.ncbi.nlm.nih.gov/genomes/all/GCF/000/686/705/GCF_000686705.1_ASM68670v1
