# Supplementary material for: Genome sequence of the H2-producing Clostridium beijerinckii strain Br21 isolated from a sugarcane vinasse treatment plant
Source: Genet Mol Biol. 2019 Jan 31;42(1):139–44. doi: 10.1590/1678-4685-GMB-2017-0315 (PMC6428130; doi:10.1590/1678-4685-GMB-2017-0315)
Supplement: Supplementary file 5 [file 1415-4757-GMB-1678-4685-GMB-2017-0315-s004.pdf]

Supplementary Material "Genome sequence of the H_2-producing *Clostridium beijerinckii* strain Br21 isolated from a sugarcane vinasse treatment plant"

Table S3 - Complete results from the Genome-to-Genome Distance Calculator and OrthoANIu.

Query Species name	Query Species AC	Reference Species name	Reference Species AC	Formula 1 (HSP length / total length)				Formula 2 (identities / HSP length)				Formula 3 (identities / total length)			
				Distance	DDH estimate (GLM-based) [Confidence interval]	Probability that DDH > 70% (i.e., same species)	Probability that DDH > 79% (i.e., same subspecies)	Distance	DDH estimate (GLM-based) [Confidence interval]	Probability that DDH > 70% (i.e., same species)	Probability that DDH > 79% (i.e., same subspecies)	Distance	DDH estimate (GLM-based) [Confidence interval]	Probability that DDH > 70% (i.e., same species)	Probability that DDH > 79% (i.e., same subspecies)
Clostridium beijerinckii strain Br21	MWMH01.1	*Clostridium cellulovorans* 743B	NC_014393.1	0.9812	12.80% [10.1 - 16.1%]	0.00%	0.00%	0.1939	22.60% [20.3 - 25%]	0.00%	0.00%	0.9848	13.20% [10.9 - 16%]	0.00%	0.00%
Clostridium beijerinckii strain Br21	MWMH01.1	*Clostridium beijerinckii* NCIMB 14988	NZ_CP010086.2	0.2319	61.30% [57.6 - 64.9%]	46.90%	11.19%	0.0419	66.00% [63.1 - 68.8%]	69.87%	21.79%	0.2640	63.70% [60.4 - 66.9%]	43.25%	10.64%
Clostridium beijerinckii strain Br21	MWMH01.1	*Clostridium kluyveri* DSM 555	NC_009706.1	0.9863	12.70% [10.1 - 16%]	0.00%	0.00%	0.1982	22.10% [19.9 - 24.6%]	0.00%	0.00%	0.9890	13.10% [10.8 - 15.9%]	0.00%	0.00%

Query Species name	Query Species AC	Reference Species name	Reference Species AC	Formula 1 (HSP length / total length)				Formula 2 (identities / HSP length)				Formula 3 (identities / total length)			
				Distance	DDH estimate (GLM-based) [Confidence interval]	Probability that DDH > 70% (i.e., same species)	Probability that DDH > 79% (i.e., same subspecies)	Distance	DDH estimate (GLM-based) [Confidence interval]	Probability that DDH > 70% (i.e., same species)	Probability that DDH > 79% (i.e., same subspecies)	Distance	DDH estimate (GLM-based) [Confidence interval]	Probability that DDH > 70% (i.e., same species)	Probability that DDH > 79% (i.e., same subspecies)
Clostridium beijerinckii strain Br21	MWMH01.1	*Clostridium beijerinckii* NCIMB 8052	NC_009617.1	0.2118	64.80% [61 - 68.4%]	59.09%	17.39%	0.0429	65.30% [62.4 - 68.2%]	68.15%	20.67%	0.2456	66.70% [63.3 - 70%]	59.20%	15.93%
Clostridium beijerinckii strain Br21	MWMH01.1	*Clostridium acetobutylicum* ATCC 824	NC_003030.1	0.9792	12.90% [10.2 - 16.2%]	0.00%	0.00%	0.2012	21.80% [19.6 - 24.2%]	0.00%	0.00%	0.9834	13.20% [10.9 - 16%]	0.00%	0.00%
Clostridium beijerinckii strain Br21	MWMH01.1	*Clostridium butyricum* strain TOA	NZ_CP014704.1	0.8925	14.70% [11.8 - 18.1%]	0.00%	0.00%	0.1897	23.10% [20.8 - 25.5%]	0.00%	0.00%	0.9129	14.90% [12.4 - 17.8%]	0.00%	0.00%
Clostridium beijerinckii strain Br21	MWMH01.1	*Clostridium beijerinckii* ATCC 35702	NZ_CP006777.1	0.2120	64.80% [61 - 68.4%]	58.95%	17.31%	0.0429	65.30% [62.4 - 68.1%]	68.13%	20.66%	0.2459	66.70% [63.3 - 69.9%]	59.00%	15.85%
Clostridium beijerinckii	MWMH01.1	*Clostridium butyricum* KNU-L09	NZ_CP013489.1	0.9897	12.70% [10 - 15.9%]	0.00%	0.00%	0.1695	25.60% [23.3 - 28.1%]	0.01%	0.01%	0.9915	13.10% [10.7 - 15.8%]	0.00%	0.00%

Query Species name	Query Species AC	Reference Species name	Reference Species AC	Formula 1 (HSP length / total length)				Formula 2 (identities / HSP length)				Formula 3 (identities / total length)			
				Distance	DDH estimate (GLM-based) [Confidence interval]	Probability that DDH > 70% (i.e., same species)	Probability that DDH > 79% (i.e., same subspecies)	Distance	DDH estimate (GLM-based) [Confidence interval]	Probability that DDH > 70% (i.e., same species)	Probability that DDH > 79% (i.e., same subspecies)	Distance	DDH estimate (GLM-based) [Confidence interval]	Probability that DDH > 70% (i.e., same species)	Probability that DDH > 79% (i.e., same subspecies)
strain Br21															
Clostridium beijerinckii strain Br21	MWMH01.1	*Clostridium beijerinckii* NRRL B-598	NZ_CP011966.2	0.2050	66.00% [62.2 - 69.7%]	63.05%	20.04%	0.0422	65.80% [62.8 - 68.6%]	69.29%	21.41%	0.2386	67.90% [64.5 - 71.2%]	65.00%	18.45%
Clostridium beijerinckii strain Br21	MWMH01.1	*Clostridium diolis* DSM 15410	NZ_AQQG00000000.1	0.1951	67.80% [64 - 71.5%]	68.49%	24.39%	0.0414	66.30% [63.4 - 69.2%]	70.66%	22.33%	0.2285	69.70% [66.2 - 72.9%]	72.59%	22.61%
Clostridium beijerinckii strain Br21	MWMH01.1	*Clostridium tetani* E88	NC_004557.1	0.9830	12.80% [10.1 - 16.1%]	0.00%	0.00%	0.2109	20.80% [18.6 - 23.2%]	0.00%	0.00%	0.9866	13.20% [10.8 - 15.9%]	0.00%	0.00%
Clostridium beijerinckii strain Br21	MWMH01.1	*Clostridium carboxidivorans* P7	NZ_CP011803.1	0.9714	13.00% [10.3 - 16.3%]	0.00%	0.00%	0.1887	23.20% [20.9 - 25.6%]	0.00%	0.00%	0.9768	13.40% [11 - 16.2%]	0.00%	0.00%
Clostri	MWMH01.1	*Clostridium*	NZ_CP009268.	0.982	12.80%	0.00%	0.00%	0.186	23.40%	0.00%	0.00%	0.985	13.20%	0.00%	0.00%

Query Species name	Query Species AC	Reference Species name	Reference Species AC	Formula 1 (HSP length / total length)				Formula 2 (identities / HSP length)				Formula 3 (identities / total length)			
				Distance	DDH estimate (GLM-based) [Confidence interval]	Probability that DDH > 70% (i.e., same species)	Probability that DDH > 79% (i.e., same subspecies)	Distance	DDH estimate (GLM-based) [Confidence interval]	Probability that DDH > 70% (i.e., same species)	Probability that DDH > 79% (i.e., same subspecies)	Distance	DDH estimate (GLM-based) [Confidence interval]	Probability that DDH > 70% (i.e., same species)	Probability that DDH > 79% (i.e., same subspecies)
dium beijerinckii strain Br21		*pasteurianum* DSM 525	1	6	[10.1 - 16.1%]			9	[21.1 - 25.9%]			9	[10.8 - 16%]		
Clostridium beijerinckii strain Br21	MWMH01.1	*Clostridium saccharobutylicum* DSM 13864	NC_022571.1	0.7944	17.30% [14.2 - 20.8%]	0.00%	0.00%	0.1678	25.90% [23.5 - 28.4%]	0.01%	0.01%	0.8289	17.30% [14.7 - 20.3%]	0.00%	0.00%
Clostridium beijerinckii strain Br21	MWMH01.1	*Clostridium butyricum* strain JKY6D1	NZ_CP013352.1	0.8933	14.70% [11.8 - 18.1%]	0.00%	0.00%	0.1884	23.20% [20.9 - 25.7%]	0.00%	0.00%	0.9134	14.90% [12.4 - 17.8%]	0.00%	0.00%
Clostridium beijerinckii strain Br21	MWMH01.1	*Clostridium butyricum* KNU-L09	NZ_CP013252.1	0.8910	14.70% [11.9 - 18.1%]	0.00%	0.00%	0.1873	23.40% [21.1 - 25.8%]	0.00%	0.00%	0.9114	15.00% [12.5 - 17.8%]	0.00%	0.00%
Clostridium beijerinckii strain	MWMH01.1	*Clostridium butyricum* CDC_51208	NZ_CP013239.1	0.8919	14.70% [11.8 - 18.1%]	0.00%	0.00%	0.1888	23.20% [20.9 - 25.6%]	0.00%	0.00%	0.9123	14.90% [12.5 - 17.8%]	0.00%	0.00%

Query Species name	Query Species AC	Reference Species name	Reference Species AC	Formula 1 (HSP length / total length)				Formula 2 (identities / HSP length)				Formula 3 (identities / total length)			
				Distance	DDH estimate (GLM-based) [Confidence interval]	Probability that DDH > 70% (i.e., same species)	Probability that DDH > 79% (i.e., same subspecies)	Distance	DDH estimate (GLM-based) [Confidence interval]	Probability that DDH > 70% (i.e., same species)	Probability that DDH > 79% (i.e., same subspecies)	Distance	DDH estimate (GLM-based) [Confidence interval]	Probability that DDH > 70% (i.e., same species)	Probability that DDH > 79% (i.e., same subspecies)
Br21															
Clostridium beijerinckii strain Br21	MWMH01.1	*Clostridium beijerinckii* NRRL B-528	NZ_LZZK00000000.1	0.2135	64.50% [60.7 - 68.1%]	58.09%	16.78%	0.0273	76.70% [73.7 - 79.4%]	87.63%	41.45%	0.2350	68.50% [65.1 - 71.8%]	67.80%	19.85%
Clostridium beijerinckii strain Br21	MWMH01.1	*Clostridium beijerinckii* NCIMB 8052	GCF_000016965.1	0.2118	64.80% [61 - 68.4%]	59.09%	17.39%	0.0429	65.30% [62.4 - 68.2%]	68.15%	20.67%	0.2456	66.70% [63.3 - 70%]	59.20%	15.93%
Clostridium beijerinckii strain Br21	MWMH01.1	[Clostridium] cellulolyticum H10	NC_011898.1	0.9952	12.60% [9.9 - 15.8%]	0.00%	0.00%	0.1842	23.70% [21.4 - 26.2%]	0.00%	0.00%	0.9961	13.00% [10.6 - 15.7%]	0.00%	0.00%
Clostridium beijerinckii strain Br21	MWMH01.1	Clostridium aerotolerans DSM 5434	JHWJ00000000.1	0.9988	12.50% [9.8 - 15.8%]	0.00%	0.00%	0.1627	26.60% [24.3 - 29.1%]	0.02%	0.01%	0.9990	12.90% [10.6 - 15.7%]	0.00%	0.00%
Clostridium	MWMH01.1	Clostridium saccharolyticum	NC_014376.1	0.9978	12.50% [9.9 -	0.00%	0.00%	0.1644	26.40% [24 -	0.02%	0.01%	0.9982	12.90% [10.6 -	0.00%	0.00%

Query Species name	Query Species AC	Reference Species name	Reference Species AC	Formula 1 (HSP length / total length)				Formula 2 (identities / HSP length)				Formula 3 (identities / total length)			
				Distance	DDH estimate (GLM-based) [Confidence interval]	Probability that DDH > 70% (i.e., same species)	Probability that DDH > 79% (i.e., same subspecies)	Distance	DDH estimate (GLM-based) [Confidence interval]	Probability that DDH > 70% (i.e., same species)	Probability that DDH > 79% (i.e., same subspecies)	Distance	DDH estimate (GLM-based) [Confidence interval]	Probability that DDH > 70% (i.e., same species)	Probability that DDH > 79% (i.e., same subspecies)
beijerinckii strain Br21		WM1			15.8%]				28.8%]				15.7%]		
Clostridium beijerinckii strain Br21	MWMH01.1	Clostridium tyrobutyricum ASM164265v1	NZ_CP014170.1	0.9864	12.70% [10.1 - 16%]	0.00%	0.00%	0.2088	21.00% [18.8 - 23.5%]	0.00%	0.00%	0.9892	13.10% [10.8 - 15.9%]	0.00%	0.00%
Clostridium beijerinckii strain Br21	MWMH01.1	Clostridium hydrogeniformans DSM 21757	JMLJ00000000.1	0.9860	12.70% [10.1 - 16%]	0.00%	0.00%	0.2283	19.30% [17.1 - 21.6%]	0.00%	0.00%	0.9892	13.10% [10.8 - 15.9%]	0.00%	0.00%
Clostridium beijerinckii strain Br21	MWMH01.1	Clostridium thermobutyricum DSM 4928	LTAY00000000.1	0.9575	13.30% [10.6 - 16.6%]	0.00%	0.00%	0.2130	20.60% [18.4 - 23%]	0.00%	0.00%	0.9665	13.60% [11.2 - 16.4%]	0.00%	0.00%
Clostridium beijerinckii strain Br21	MWMH01.1	Clostridium colicanis 209318	LTBB00000000.1	0.9818	12.80% [10.1 - 16.1%]	0.00%	0.00%	0.1926	22.70% [20.5 - 25.2%]	0.00%	0.00%	0.9853	13.20% [10.9 - 16%]	0.00%	0.00%

Query Species name	Query Species AC	Reference Species name	Reference Species AC	Formula 1 (HSP length / total length)				Formula 2 (identities / HSP length)				Formula 3 (identities / total length)			
				Distance	DDH estimate (GLM-based) [Confidence interval]	Probability that DDH > 70% (i.e., same species)	Probability that DDH > 79% (i.e., same subspecies)	Distance	DDH estimate (GLM-based) [Confidence interval]	Probability that DDH > 70% (i.e., same species)	Probability that DDH > 79% (i.e., same subspecies)	Distance	DDH estimate (GLM-based) [Confidence interval]	Probability that DDH > 70% (i.e., same species)	Probability that DDH > 79% (i.e., same subspecies)
Clostridium beijerinckii strain Br21	MWMH01.1	Clostridium saccharoperbutylacetonicum N1-4	NC_020291.1	0.7714	18.00% [14.9 - 21.5%]	0.00%	0.00%	0.1681	25.80% [23.5 - 28.3%]	0.01%	0.01%	0.8098	18.00% [15.3 - 21%]	0.00%	0.00%
Clostridium diolis DSM 15410	AQQG00000000.1	*Clostridium cellulovorans* 743B	NC_014393.1	0.9836	12.80% [10.1 - 16.1%]	0.00%	0.00%	0.1856	23.60% [21.3 - 26%]	0.00%	0.00%	0.9867	13.20% [10.8 - 15.9%]	0.00%	0.00%
Clostridium diolis DSM 15410	AQQG00000000.1	*Clostridium butyricum* TOA	NZ_CP014704.1	0.9011	14.50% [11.6 - 17.9%]	0.00%	0.00%	0.1858	23.50% [21.2 - 26%]	0.00%	0.00%	0.9195	14.70% [12.3 - 17.6%]	0.00%	0.00%
Clostridium diolis DSM 15410	AQQG00000000.1	*Clostridium beijerinckii* ATCC 35702	NZ_CP006777.1	0.1404	78.40% [74.4 - 81.9%]	89.27%	56.67%	0.0244	79.20% [76.2 - 81.8%]	89.91%	46.15%	0.1614	81.40% [78 - 84.4%]	96.52%	61.33%
Clostridium diolis DSM 15410	AQQG00000000.1	*Clostridium beijerinckii* NCIMB 14988	NZ_CP010086.2	0.1752	71.60% [67.6 - 75.2%]	77.99%	34.93%	0.0280	76.20% [73.2 - 79%]	87.10%	40.49%	0.1983	74.90% [71.4 - 78.1%]	88.38%	38.46%
Clostri	AQQG0000	*Clostridium*	NC_009706.1	0.987	12.70%	0.00%	0.00%	0.192	22.80%	0.00%	0.00%	0.990	13.10%	0.00%	0.00%

Query Species name	Query Species AC	Reference Species name	Reference Species AC	Formula 1 (HSP length / total length)				Formula 2 (identities / HSP length)				Formula 3 (identities / total length)			
				Distance	DDH estimate (GLM-based) [Confidence interval]	Probability that DDH > 70% (i.e., same species)	Probability that DDH > 79% (i.e., same subspecies)	Distance	DDH estimate (GLM-based) [Confidence interval]	Probability that DDH > 70% (i.e., same species)	Probability that DDH > 79% (i.e., same subspecies)	Distance	DDH estimate (GLM-based) [Confidence interval]	Probability that DDH > 70% (i.e., same species)	Probability that DDH > 79% (i.e., same subspecies)
dium diolis DSM 15410	0000.1	*kluyveri* DSM 555		6	[10 - 16%]			4	[20.5 - 25.2%]			0	[10.8 - 15.9%]		
Clostridium diolis DSM 15410	AQQG00000000.1	*Clostridium beijerinckii* NCIMB 8052	NC_009617.1	0.1402	78.40% [74.5 - 81.9%]	89.32%	56.82%	0.0244	79.20% [76.2 - 81.8%]	89.91%	46.16%	0.1612	81.40% [78.1 - 84.4%]	96.54%	61.47%
Clostridium diolis DSM 15410	AQQG00000000.1	*Clostridium acetobutylicum* ATCC 824	NC_003030.1	0.9769	12.90% [10.2 - 16.2%]	0.00%	0.00%	0.2051	21.40% [19.2 - 23.8%]	0.00%	0.00%	0.9817	13.30% [10.9 - 16.1%]	0.00%	0.00%
Clostridium diolis DSM 15410	AQQG00000000.1	*Clostridium butyricum* KNU-L09	NZ_CP013489.1	0.9889	12.70% [10 - 16%]	0.00%	0.00%	0.1962	22.30% [20.1 - 24.8%]	0.00%	0.00%	0.9911	13.10% [10.7 - 15.8%]	0.00%	0.00%
Clostridium diolis DSM 15410	AQQG00000000.1	*Clostridium beijerinckii* NRRL B-598	NZ_CP011966.2	0.1096	84.50% [80.8 - 87.7%]	94.66%	74.24%	0.0136	88.60% [86.2 - 90.7%]	95.31%	63.04%	0.1217	88.10% [85.1 - 90.5%]	99.11%	81.18%
Clostridium diolis	AQQG00000000.1	*Clostridium pasteurianum* DSM 525	NZ_CP009268.1	0.9806	12.80% [10.1 - 16.1%]	0.00%	0.00%	0.1906	23.00% [20.7 - 25.4%]	0.00%	0.00%	0.9843	13.20% [10.9 - 16%]	0.00%	0.00%

Query Species name	Query Species AC	Reference Species name	Reference Species AC	Formula 1 (HSP length / total length)				Formula 2 (identities / HSP length)				Formula 3 (identities / total length)			
				**Distance**	**DDH estimate (GLM-based) [Confidence interval]**	**Probability that DDH > 70% (i.e., same species)**	**Probability that DDH > 79% (i.e., same subspecies)**	**Distance**	**DDH estimate (GLM-based) [Confidence interval]**	**Probability that DDH > 70% (i.e., same species)**	**Probability that DDH > 79% (i.e., same subspecies)**	**Distance**	**DDH estimate (GLM-based) [Confidence interval]**	**Probability that DDH > 70% (i.e., same species)**	**Probability that DDH > 79% (i.e., same subspecies)**
DSM 15410															
Clostridium diolis DSM 15410	AQQG00000000.1	*Clostridium saccharobutylicum* DSM 13864	NC_022571.1	0.7986	17.10% [14.1 - 20.7%]	0.00%	0.00%	0.1662	26.10% [23.8 - 28.6%]	0.02%	0.01%	0.8321	17.20% [14.6 - 20.2%]	0.00%	0.00%
Clostridium diolis DSM 15410	AQQG00000000.1	*Clostridium butyricum* JKY6D1	NZ_CP013352.1	0.9009	14.50% [11.6 - 17.9%]	0.00%	0.00%	0.1848	23.60% [21.3 - 26.1%]	0.00%	0.00%	0.9192	14.80% [12.3 - 17.6%]	0.00%	0.00%
Clostridium diolis DSM 15410	AQQG00000000.1	*Clostridium butyricum* CDC_51208	NZ_CP013239.1	0.8998	14.50% [11.7 - 17.9%]	0.00%	0.00%	0.1823	24.00% [21.7 - 26.4%]	0.00%	0.00%	0.9181	14.80% [12.3 - 17.6%]	0.00%	0.00%
Clostridium diolis DSM 15410	AQQG00000000.1	Clostridium carboxidivorans P7	NZ_CP011803.1	0.9692	13.10% [10.3 - 16.4%]	0.00%	0.00%	0.1872	23.40% [21.1 - 25.8%]	0.00%	0.00%	0.9749	13.40% [11.1 - 16.2%]	0.00%	0.00%
Clostridium diolis DSM 15410	AQQG00000000.1	[Clostridium] cellulolyticum H10	NC_011898.1	0.9960	12.60% [9.9 - 15.8%]	0.00%	0.00%	0.1613	26.80% [24.5 - 29.3%]	0.02%	0.01%	0.9966	13.00% [10.6 - 15.7%]	0.00%	0.00%

Query Species name	Query Species AC	Reference Species name	Reference Species AC	Formula 1 (HSP length / total length)				Formula 2 (identities / HSP length)				Formula 3 (identities / total length)			
				Distance	DDH estimate (GLM-based) [Confidence interval]	Probability that DDH > 70% (i.e., same species)	Probability that DDH > 79% (i.e., same subspecies)	Distance	DDH estimate (GLM-based) [Confidence interval]	Probability that DDH > 70% (i.e., same species)	Probability that DDH > 79% (i.e., same subspecies)	Distance	DDH estimate (GLM-based) [Confidence interval]	Probability that DDH > 70% (i.e., same species)	Probability that DDH > 79% (i.e., same subspecies)
Clostridium diolis DSM 15410	AQQG00000000.1	Clostridium aerotolerans DSM 5434	JHWJ00000000.1	0.9980	12.50% [9.9 - 15.8%]	0.00%	0.00%	0.1302	32.30% [29.9 - 34.8%]	0.26%	0.10%	0.9982	12.90% [10.6 - 15.7%]	0.00%	0.00%
Clostridium diolis DSM 15410	AQQG00000000.1	Clostridium saccharolyticum WM1	NC_014376.1	0.9982	12.50% [9.9 - 15.8%]	0.00%	0.00%	0.1624	26.70% [24.3 - 29.1%]	0.02%	0.01%	0.9985	12.90% [10.6 - 15.7%]	0.00%	0.00%
Clostridium diolis DSM 15410	AQQG00000000.1	Clostridium tyrobutyricum ASM164265v1	NZ_CP014170.1	0.9868	12.70% [10 - 16%]	0.00%	0.00%	0.1976	22.20% [19.9 - 24.6%]	0.00%	0.00%	0.9894	13.10% [10.8 - 15.9%]	0.00%	0.00%
Clostridium diolis DSM 15410	AQQG00000000.1	Clostridium hydrogeniformans DSM 21757	JMLJ00000000.1	0.9860	12.70% [10.1 - 16%]	0.00%	0.00%	0.2359	18.60% [16.5 - 21%]	0.00%	0.00%	0.9893	13.10% [10.8 - 15.9%]	0.00%	0.00%
Clostridium diolis DSM 15410	AQQG00000000.1	Clostridium thermobutyricum DSM 4928	LTAY00000000.1	0.9611	13.20% [10.5 - 16.5%]	0.00%	0.00%	0.2133	20.60% [18.4 - 23%]	0.00%	0.00%	0.9694	13.60% [11.2 - 16.3%]	0.00%	0.00%
Clostridium	AQQG00000000.1	Clostridium colicanis 209318	LTBB00000000.1	0.9796	12.90% [10.2 -	0.00%	0.00%	0.1911	22.90% [20.6 -	0.00%	0.00%	0.9835	13.20% [10.9 -	0.00%	0.00%

Query Species name	Query Species AC	Reference Species name	Reference Species AC	Formula 1 (HSP length / total length)				Formula 2 (identities / HSP length)				Formula 3 (identities / total length)			
				Distance	DDH estimate (GLM-based) [Confidence interval]	Probability that DDH > 70% (i.e., same species)	Probability that DDH > 79% (i.e., same subspecies)	Distance	DDH estimate (GLM-based) [Confidence interval]	Probability that DDH > 70% (i.e., same species)	Probability that DDH > 79% (i.e., same subspecies)	Distance	DDH estimate (GLM-based) [Confidence interval]	Probability that DDH > 70% (i.e., same species)	Probability that DDH > 79% (i.e., same subspecies)
diolis DSM 15410					16.1%]				25.4%]				16%]		
Clostridium diolis DSM 15410	AQQG00000000.1	Clostridium saccharoperbutylacetonicum N1-4	NC_020291.1	0.7632	18.20% [15.1 - 21.8%]	0.00%	0.00%	0.1685	25.80% [23.4 - 28.2%]	0.01%	0.01%	0.8031	18.20% [15.5 - 21.2%]	0.00%	0.00%
Clostridium diolis DSM 15410	AQQG00000000.1	*Clostridium tetani* E88	NC_004557.1	0.9827	12.80% [10.1 - 16.1%]	0.00%	0.00%	0.2076	21.10% [18.9 - 23.6%]	0.00%	0.00%	0.9863	13.20% [10.8 - 15.9%]	0.00%	0.00%
