# Supplementary material for: Genome sequence of the H2-producing Clostridium beijerinckii strain Br21 isolated from a sugarcane vinasse treatment plant
Source: Genet Mol Biol. 2019 Jan 31;42(1):139–44. doi: 10.1590/1678-4685-GMB-2017-0315 (PMC6428130; doi:10.1590/1678-4685-GMB-2017-0315)
Supplement: Supplementary file 6 [file 1415-4757-GMB-1678-4685-GMB-2017-0315-s001.pdf]

Supplementary Material "Genome sequence of the H_2-producing *Clostridium beijerinckii* strain Br21 isolated from a sugarcane vinasse treatment plant"

Extended description of Br21 strain taxonomic assignment

Extended description of Br21 strain taxonomic assignment 1
Brief description of strain isolation 1
Strain identification by 16S rDNA sequencing and phylogeny 1
Multilocus phylogeny 2
Prediction of DNA-DNA hybridization values 3
Average Nucleotide Identity 3

Brief description of strain isolation

We refer the readers to our previous work (https://doi.org/10.1016/j.ijhydene.2016.09.110) where we extensively describe the isolation of strain Br21 from an upflow anaerobic sludge blanked reactor employed to treat wastewater from a sugar mill and ethanol industry. Briefly, after pre-treatment to enrich for spore-forming bacteria 100 µL of sludge was inoculated in CH medium, specific for the cultivation of *Clostridium* species, growth was allowed at 35 ºC in an Anaerojar (Oxoid® AG025A) with a pack of Anaerogen (Oxoid® AN025A) until colonies emerged.

Strain identification by 16S rDNA sequencing and phylogeny

After genome assembly, the full 16S rRNA gene was extracted from the genome sequence. The 16S rDNA was used as query to search for similar sequences using the "Identify" tool from the EzBiocloud (https://www.ezbiocloud.net/). Only sequences with at least 94% identity to the query, and originating from type strains with names standing in nomenclature were kept for phylogenetic analysis. Table S1 shows the list of all the sequences kept in this first step, as well as percent identity to the query sequence, and number of nucleotide differences. It is important to note that even at this step, there was a strong suggestion that the strain Br21 belongs to either *C. diolis* or *C. beijerinckii*, with 99.8% nucleotide identity and only three nucleotide substitutions to either of them. It is also important to note that all species detected in this first step belong to the genus *Clostridium*, the most divergent with an identity of 94.8% with 73 nucleotide substitutions in its 16S rDNA compared to Br21.

In a second step, all the sequences gathered during the first step were used for phylogenetic inference. A multiple sequence alignment was carried out with MAFFT v7.220 using the Q-INS-i option, which takes into account secondary structure, first folding the sequences and then aligning them keeping into account the secondary structure (https://doi.org/10.1186/1471-2105-9-212). Phylogenetic inference was carried out using RAxML v8.2.11 under the GTR+Γ+I with automatic bootstrapping. The resulting phylogenetic tree was visualized with FigTree v1.4.2 and rooted on the midpoint. Figure 2 in the main text of the manuscript clearly shows Br21 (in bold face in the tree) 16S rDNA sequence clustering with that of *C. diolis* and *C. beijerinckii* on a clade with 51% bootstrap support.

Due to the low bootstrap support on the clade where Br21 is located obtained in the 16S rDNA phylogeny, we decided to exploit the whole genome sequence data to improve the phylogenetic analysis. For this we used three complementary approaches. First, inference of a multilocus phylogeny. Second, the prediction of DNA-DNA hybridization values. Third, the computation of the Average Nucleotide Identity (ANI) among orthologous genes between the species/genomes of interest.

Multilocus phylogeny

We used Phyla AMPHORA (https://doi.org/10.1093/molbev/mst059) to identify a set of 168 phylogenetic markers among the phyla Firmicutes. These are single-copy bacterial genes, with low evidence of lateral gene transfer. The full genome sequence of the strains listed in Table S2 was downloaded from NCBI. Phyla AMPHORA provides a Hidden Markov Model (HMM) file for each of the markers. HMMs are used with the `MarkerScanner.pl` script to query the genome of target species for the marker of interest, the results of which are used by `MarkerAlignTrim.pl` to produce a multiple sequence alignment for each marker gene (both scripts are provided by the Phyla AMPHORA pipeline).

The multiple amino acid sequence alignments for each marker gene were concatenated into a super matrix using FASconCAT-G (https://doi.org/10.1186/s12983-014-0081-x). The proper evolutionary model for phylogenetic inference was chosen for each marker gene using ProtTest3 (https://doi.org/10.1093/bioinformatics/btr088), and phylogenetic analysis was carried out with RAxML (https://doi.org/10.1093/bioinformatics/btu033) with automatic bootstrapping and using a different partition for each marker gene and its evolutionary model: 146 genes (partitions in the supermatrix) were analyzed under the LG model, 15 under CPREV, 3 under FLU, and 4 in each of MTART, DAYHOFF, WAG and JTT (Dataset S1 contains single maker multiple sequence alignments, evolutionary models, supermatrix and phylogenetic tree in newick format, and Figure S1 contains the final phylogenetic tree).

The multilocus phylogeny (Figure S1) clearly shows that all *C. beijerincki* strains, together with the single *C. diolis* strain form a very cohesive clade with 100% bootstrap support. Our strain

Br21 is located into this *C. beijerincki* clade. Even more, Br21 forms a separate clade within *C. beijerincki* together with the strains: DSM53, NRRL B-593 and NRRL B-528. These results provide additional evidence that Br21 is a strain within the *C. beijerincki* species.

Prediction of DNA-DNA hybridization values

Before the development of next generation sequencing technologies (NGS), and associated technologies to produce complete or nearly-complete genome sequences of prokaryotic organisms, species delimitation in Archaea and Bacteria was based on DNA-DNA hybridization (DDH), according to which two organisms belong to different species if their DDH is less than 70%. DDH is very time-consuming wet-lab procedure that required specially trained personnel, and even then it is prone to error. More recently, following the decrease in cost and effort to obtain a high quality bacterial genome assembly, different computational methods have appeared to replace the wet-lab DDH. One of these computational methods is the Genome-to-Genome Distance Calculator (GGDC; http://ggdc.dsmz.de/; https://doi.org/10.1186/1471-2105-14-60) that can compare any pair of prokaryotic genome sequences and provide digital measure of DDH, that are highly correlated to wet-lab DDH values, and that can be used in the same fashion and with the same thresholds for species and sub-species level assignments. We used the GGDH tools to compute digital DDH for representatives of the species listed in Table S3, and the GGDC results are available in Table S1. In Table S1 pay attention to the results for “Formula 2” which is the recommended in the case of incomplete genome, and which is independent of the genome length. The Formula 2 of GGDC also shows that Br21 is closely related to *C. beijerincki* and *C. diolis* strains, with DDH values going from 65.30% to 76.70%, more than to any other of the *Clostridium* species tested. With DDH, 70% similarity is taken as strong evidence that the two genomes being compared belong to the same species. The highest digital DDH was against *C. beijerincki* NRRL B-528, and it is actually the only value above the 70% threshold. It can also be noted in Table S1, that the single *C. diolis* strain with a genome sequence available have digital DDH values always over 70% when compared to *C. beijerincki* strains, even reaching values up to 86.2%. Digital DDH values above 79% had been recently suggested as evidence that the two organisms being compared belong to the same sub-species.

Average Nucleotide Identity

Another alternative to wet-lab DDH that tries to leverage genome information is the computation of the Average Nucleotide Identity (ANI), and particularly more recently computing the ANI among orthologous genes between two genomes that are being compared (https://doi.org/10.1099/ijsem.0.000760). ANI approaches have also been proposed to completely overhaul prokaryotic systematics in the post-genomics era

(https://doi.org/10.1093/nar/gkv657). Here we decided to employ OrthoANIu, and approach that uses orthologous genome fragments for the computation of the ANI, that is genome fragments that are their reciprocal best hits in BLASTn searches. The genome wide ANI is calculated as the average of the identity values of all orthologous genome fragments between the two organisms being compared (https://doi.org/10.1099/ijsem.0.000760). Species level demarcation have been recommend at ANI values of 95-96% (https://doi.org/10.1099/ijsem.0.000760 and reference therein) and at 95.6% (https://doi.org/10.1093/nar/gkv657). We applied OrthoANIu to compare the genome of our species of interest Br21 against the available genomes of all *C. beijerincki* and *C. diolis* strains (Table S1). Br21 have ANI values greater than 96% to all C. beijerincki and C. diolis strains. It has an ANI larger than 96.5% against only three strains: *C. beijerinckii* DSM 53, *C. beijerinckii* NRRL B-593, *C. beijerinckii* NRRL B-528
