# Supplementary material for: Genome sequence of the H2-producing Clostridium beijerinckii strain Br21 isolated from a sugarcane vinasse treatment plant
Source: Genet Mol Biol. 2019 Jan 31;42(1):139–44. doi: 10.1590/1678-4685-GMB-2017-0315 (PMC6428130; doi:10.1590/1678-4685-GMB-2017-0315)
Supplement: Supplementary file 7 [file 1415-4757-GMB-1678-4685-GMB-2017-0315-s005.pdf]

# Supplementary Material "Genome sequence of the $H_2$-producing *Clostridium beijerinckii* strain Br21 isolated from a sugarcane vinasse treatment plant"

**Table S4** - Genes encoding for glycosyl hydrolases identified in the *C. beijerinckii* strain Br21 genome.

| Protein Accession Number | Feature ID (RAST) | Type | Contig | Start | Stop | Frame | Strand | Length (bp) | Function | Subsystem |
|---|---|---|---|---|---|---|---|---|---|---|
| OOP74710.1 | fig\|1520.77.peg.1451 | CDS | NODE_1_length_1193148_cov_98.3687 | 149584 | 151026 | 1 | + | 1443 | Beta-glucosidase (EC 3.2.1.21) | 6-phospho-beta-glucosidase (EC 3.2.1.86) |
| OOP72390.1 | fig\|1520.77.peg.4118 | CDS | NODE_5_length_413361_cov_65.9822 | 178024 | 178545 | 1 | + | 522 | Isochorismatase (EC 3.3.2.1) | Chorismate: Intermediate for synthesis of Tryptophan |
| OOP70845.1 | fig\|1520.77.peg.488 | CDS | NODE_12_length_157230_cov_67.3277 | 133860 | 135305 | 3 | + | 1446 | 6-phospho-beta-glucosidase (EC 3.2.1.86) | Beta-Glucoside Metabolism |
| OOP74869.1 | fig\|1520.77.peg.1629 | CDS | NODE_1_length_1193148_cov_98.3687 | 399717 | 401177 | 3 | + | 1461 | 6-phospho-beta-glucosidase (EC 3.2.1.86) | Beta-Glucoside Metabolism |
| OOP75463.1 | fig\|1520.77.peg.1304 | CDS | NODE_1_length_1193148_cov_98.3687 | 1165904 | 1167337 | 2 | + | 1434 | 6-phospho-beta-glucosidase (EC 3.2.1.86) | Beta-Glucoside Metabolism |
| OOP74867.1 | fig\|1520.77.peg.1626 | CDS | NODE_1_length_1193148_cov_98.3687 | 397182 | 398543 | 3 | + | 1362 | 6-phospho-beta-glucosidase (EC 3.2.1.86) | Beta-Glucoside Metabolism |
| OOP75404.1 | fig\|1520.77.peg.1236 | CDS | NODE_1_length_1193148_cov_98.3687 | 1091588 | 1092988 | 2 | + | 1398 | 6-phospho-beta-glucosidase (EC 3.2.1.86) | Beta-Glucoside Metabolism |
| OOP74699.1 | fig\|1520.77.peg.1440 | CDS | NODE_1_length_1193148_cov_98.3687 | 135846 | 137234 | 3 | + | 1389 | 6-phospho-beta-glucosidase (EC 3.2.1.86) | Beta-Glucoside Metabolism |
| OOP74869.1 | fig\|1520.77.peg.1627 | CDS | NODE_1_length_1193148_cov_98.3687 | 398573 | 398716 | 2 | + | 144 | 6-phospho-beta-glucosidase (EC 3.2.1.86) | Beta-Glucoside Metabolism |
| OOP74894.1 | fig\|1520.77.peg.1655 | CDS | NODE_1_length_1193148_cov_98.3687 | 437765 | 439177 | 2 | + | 1413 | 6-phospho-beta-glucosidase (EC 3.2.1.86) | Beta-Glucoside Metabolism |
| OOP73038.1 | fig\|1520.77.peg.3876 | CDS | NODE_4_length_604572_cov_120.866 | 520929 | 522353 | 3 | + | 1425 | 6-phospho-beta-glucosidase ascB (EC 3.2.1.86) | Beta-Glucoside Metabolism |
| OOP71843.1 | fig\|1520.77.peg.4951 | CDS | NODE_7_length_339772_cov_75.5879 | 258636 | 256516 | -3 | - | 2121 | Alpha-galactosidase (EC 3.2.1.22) | Fructooligosaccharides(FOS) and Raffinose Utilization |
| NA | fig\|1520.77.peg.1757 | CDS | NODE_1_length_1193148_cov_98.3687 | 557559 | 558062 | 3 | + | 504 | 6-phospho-beta-glucosidase (EC 3.2.1.86) | Beta-Glucoside Metabolism |

| Protein Accession Number | Feature ID (RAST) | Type | Contig | Start | Stop | Frame | Strand | Length (bp) | Function | Subsystem |
|---|---|---|---|---|---|---|---|---|---|---|
| OOP73321.1 | fig\|1520.77.peg.2889 | CDS | NODE_3_length_819615_cov_56.6944 | 260188 | 261498 | 1 | + | 1311 | 6-phospho-beta-glucosidase (EC 3.2.1.86) | Beta-Glucoside Metabolism |
| OOP75093.1 | fig\|1520.77.peg.898 | CDS | NODE_1_length_1193148_cov_98.3687 | 698921 | 697599 | -2 | - | 1323 | Alpha-galactosidase (EC 3.2.1.22) | Fructooligosaccharides(FOS) and Raffinose Utilization |
| OOP74882.1 | fig\|1520.77.peg.1642 | CDS | NODE_1_length_1193148_cov_98.3687 | 419304 | 421814 | 3 | + | 2511 | Alpha-glucosidase (EC 3.2.1.20) | D-Galacturonate and D-Glucuronate Utilization |
| OOP74884.1 | fig\|1520.77.peg.1644 | CDS | NODE_1_length_1193148_cov_98.3687 | 423262 | 425751 | 1 | + | 2490 | Alpha-glucosidase (EC 3.2.1.20) | D-Galacturonate and D-Glucuronate Utilization |
| OOP74224.1 | fig\|1520.77.peg.2285 | CDS | NODE_2_length_829829_cov_83.6585 | 459004 | 461376 | 1 | + | 2373 | Alpha-glucosidase (EC 3.2.1.20) | D-Galacturonate and D-Glucuronate Utilization |
| NA | fig\|1520.77.peg.2805 | CDS | NODE_3_length_819615_cov_56.6944 | 173426 | 173746 | 2 | + | 321 | Alpha-mannosidase (EC 3.2.1.24) | Fructooligosaccharides(FOS) and Raffinose Utilization |
| OOP73604.1 | fig\|1520.77.peg.3184 | CDS | NODE_3_length_819615_cov_56.6944 | 610001 | 608484 | -2 | - | 1518 | Alpha-mannosidase (EC 3.2.1.24) | L-Arabinose utilization |
| OOP70421.1 | fig\|1520.77.peg.1828 | CDS | NODE_21_length_5527_cov_64.2445 | 2240 | 4267 | 2 | + | 2028 | Alpha-xylosidase (EC 3.2.1.-) | Xylose utilization |
| OOP73184.1 | fig\|1520.77.peg.2734 | CDS | NODE_3_length_819615_cov_56.6944 | 88354 | 90396 | 1 | + | 2043 | Alpha-xylosidase (EC 3.2.1.-) | Xylose utilization |
| OOP72239.1 | fig\|1520.77.peg.4682 | CDS | NODE_6_length_383704_cov_86.1472 | 374759 | 372402 | -2 | - | 2358 | Alpha-xylosidase (EC 3.2.1.-) | Xylose utilization |
| OOP71837.1 | fig\|1520.77.peg.4945 | CDS | NODE_7_length_339772_cov_75.5879 | 250527 | 248086 | -3 | - | 2442 | Alpha-xylosidase (EC 3.2.1.-) | Xylose utilization |
| OOP74003.1 | fig\|1520.77.peg.2049 | CDS | NODE_2_length_829829_cov_83.6585 | 215366 | 217471 | 2 | + | 2106 | Beta-galactosidase (EC 3.2.1.23) | Galactosylceramide and Sulfatide metabolism |
| OOP74007.1 | fig\|1520.77.peg.2053 | CDS | NODE_2_length_829829_cov_83.6585 | 222920 | 225946 | 2 | + | 3027 | Beta-galactosidase (EC 3.2.1.23) | Galactosylceramide and Sulfatide metabolism |
| OOP71147.1 | fig\|1520.77.peg.149 | CDS | NODE_10_length_219934_cov_78.8972 | 147474 | 148862 | 3 | + | 1389 | Beta-glucosidase (EC 3.2.1.21) | Beta-Glucoside Metabolism |
| OOP71190.1 | fig\|1520.77.peg.195 | CDS | NODE_10_length_219934_cov_78.8972 | 198726 | 200795 | 3 | + | 2070 | Beta-glucosidase (EC 3.2.1.21) | Beta-Glucoside Metabolism |
| OOP70674.1 | fig\|1520.77.peg.564 | CDS | NODE_13_length_134357_cov_111.456 | 63913 | 61784 | -1 | - | 2130 | Beta-glucosidase (EC 3.2.1.21) | Beta-Glucoside Metabolism |
| OOP75419.1 | fig\|1520.77.peg.1258 | CDS | NODE_1_length_1193148_cov_98.3687 | 1116298 | 1114034 | -1 | - | 2265 | Beta-glucosidase (EC 3.2.1.21) | Beta-Glucoside Metabolism |
| OOP70674.1 | fig\|1520.77.peg.1620 | CDS | NODE_1_length_1193148_cov_98.3687 | 389662 | 390045 | 1 | + | 384 | Beta-glucosidase (EC 3.2.1.21) | Beta-Glucoside Metabolism |

| Protein Accession Number | Feature ID (RAST) | Type | Contig | Start | Stop | Frame | Strand | Length (bp) | Function | Subsystem |
|---|---|---|---|---|---|---|---|---|---|---|
| OOP70674.1 | fig\|1520.77.peg.1622 | CDS | NODE_1_length_1193148_cov_98.3687 | 392201 | 393898 | 2 | + | 1698 | Beta-glucosidase (EC 3.2.1.21) | Beta-Glucoside Metabolism |
| OOP74965.1 | fig\|1520.77.peg.1727 | CDS | NODE_1_length_1193148_cov_98.3687 | 521194 | 522591 | 1 | + | 1398 | Beta-glucosidase (EC 3.2.1.21) | Beta-Glucoside Metabolism |
| OOP74255.1 | fig\|1520.77.peg.2318 | CDS | NODE_2_length_829829_cov_83.6585 | 499893 | 501302 | 3 | + | 1410 | Beta-glucosidase (EC 3.2.1.21) | Beta-Glucoside Metabolism |
| OOP70672.1 | fig\|1520.77.peg.562 | CDS | NODE_13_length_134357_cov_111.456 | 60508 | 58901 | -1 | - | 1608 | Beta-xylosidase (EC 3.2.1.37) | Xylose utilization |
| OOP75319.1 | fig\|1520.77.peg.1140 | CDS | NODE_1_length_1193148_cov_98.3687 | 983007 | 984665 | 3 | + | 1659 | Beta-xylosidase (EC 3.2.1.37) | Xylose utilization |
| OOP70770.1 | fig\|1520.77.peg.403 | CDS | NODE_12_length_157230_cov_67.3277 | 36334 | 35573 | -1 | - | 762 | Chitinase (EC 3.2.1.14) | Chitin and N-acetylglucosamine utilization |
| OOP73500.1 | fig\|1520.77.peg.3081 | CDS | NODE_3_length_819615_cov_56.6944 | 473412 | 475592 | 3 | + | 2181 | Glycogen debranching enzyme (EC 3.2.1.-) | Glycogen metabolism |
| OOP74801.1 | fig\|1520.77.peg.1553 | CDS | NODE_1_length_1193148_cov_98.3687 | 284461 | 286851 | 1 | + | 2391 | Maltodextrin glucosidase (EC 3.2.1.20) | Maltose and Maltodextrin Utilization |
| OOP70752.1 | fig\|1520.77.peg.384 | CDS | NODE_12_length_157230_cov_67.3277 | 16256 | 15327 | -2 | - | 930 | Membrane-bound lytic murein transglycosylase D precursor (EC 3.2.1.-) | CBSS-228410.1.peg.134 |
| OOP72208.1 | fig\|1520.77.peg.4651 | CDS | NODE_6_length_383704_cov_86.1472 | 340260 | 339625 | -3 | - | 636 | Membrane-bound lytic murein transglycosylase D precursor (EC 3.2.1.-) | CBSS-228410.1.peg.134 |
| OOP72208.1 | fig\|1520.77.peg.4651 | CDS | NODE_6_length_383704_cov_86.1472 | 340260 | 339625 | -3 | - | 636 | Membrane-bound lytic murein transglycosylase D precursor (EC 3.2.1.-) | CBSS-228410.1.peg.134 |
| OOP71594.1 | fig\|1520.77.peg.5221 | CDS | NODE_8_length_237282_cov_113.494 | 206683 | 207489 | 1 | + | 807 | Membrane-bound lytic murein transglycosylase D precursor (EC 3.2.1.-) | CBSS-228410.1.peg.134 |
| OOP74596.1 | fig\|1520.77.peg.845 | CDS | NODE_1_length_1193148_cov_98.3687 | 7281 | 9107 | 3 | + | 1827 | Neopullulanase (EC 3.2.1.135) | |
| OOP75309.1 | fig\|1520.77.peg.1130 | CDS | NODE_1_length_1193148_cov_98.3687 | 969083 | 970426 | 2 | + | 1344 | Neopullulanase (EC 3.2.1.135) | Maltose and Maltodextrin Utilization |
| OOP75310.1 | fig\|1520.77.peg.1131 | CDS | NODE_1_length_1193148_cov_98.3687 | 970526 | 972499 | 2 | + | 1974 | Pullulanase (EC 3.2.1.41) | Maltose and Maltodextrin Utilization |
| OOP71104.1 | fig\|1520.77.peg.99 | CDS | NODE_10_length_219934_cov_78.8972 | 93450 | 94937 | 3 | + | 1488 | Sucrose-6-phosphate hydrolase (EC 3.2.1.B3) | - none - |

| Protein Accession Number | Feature ID (RAST) | Type | Contig | Start | Stop | Frame | Strand | Length (bp) | Function | Subsystem |
|---|---|---|---|---|---|---|---|---|---|---|
| OOP70706.1 | fig\|1520.77.peg.599 | CDS | NODE_13_length_134357_cov_111.456 | 96572 | 95427 | -2 | - | 1146 | Sucrose-6-phosphate hydrolase (EC 3.2.1.B3) | - none - |
| OOP73647.1 | fig\|1520.77.peg.3232 | CDS | NODE_3_length_819615_cov_56.6944 | 652431 | 650779 | -3 | - | 1653 | Trehalose-6-phosphate hydrolase (EC 3.2.1.93) | Trehalose Uptake and Utilization |
