# Supplementary material for: Genome sequence of the H2-producing Clostridium beijerinckii strain Br21 isolated from a sugarcane vinasse treatment plant
Source: Genet Mol Biol. 2019 Jan 31;42(1):139–44. doi: 10.1590/1678-4685-GMB-2017-0315 (PMC6428130; doi:10.1590/1678-4685-GMB-2017-0315)
Supplement: Supplementary file 8 [file 1415-4757-GMB-1678-4685-GMB-2017-0315-s006.pdf]

Supplementary Material “Genome sequence of the H_2-producing *Clostridium beijerinckii* strain Br21 isolated from a sugarcane vinasse treatment plant”

Table S5 - Genes encoding for enzymes and electron carriers related to biofuel and chemical production identified in the *C. beijerinckii* strain Br21 genome.

Protein Accession Number	Feature ID (RAST)	Type	Contig	Start	Stop	Frame	Strand	Length (bp)	Function	Subsystem
OOP71033.1	fig|1520.77.peg.17	CDS	NODE_10_length_219934_cov_78.8972	15990	15163	-3	-	828	Ferredoxin	Soluble cytochromes and functionally related electron carriers
OOP71120.1	fig|1520.77.peg.119	CDS	NODE_10_length_219934_cov_78.8972	121230	122048	3	+	819	Ferredoxin	Soluble cytochromes and functionally related electron carriers
OOP70963.1	fig|1520.77.peg.318	CDS	NODE_11_length_183161_cov_63.1791	121280	120972	-2	-	309	Ferredoxin	2Fe-2S
OOP70636.1	fig|1520.77.peg.523	CDS	NODE_13_length_134357_cov_111.456	13105	12284	-1	-	822	Ferredoxin	Soluble cytochromes and functionally related electron carriers
OOP70571.1	fig|1520.77.peg.676	CDS	NODE_14_length_120831_cov_113.702	52741	52550	-1	-	192	Ferredoxin	Soluble cytochromes and functionally related electron carriers
OOP74708.1	fig|1520.77.peg.1449	CDS	NODE_1_length_1193148_cov_98.3687	147027	148133	3	+	1107	Ferredoxin	Soluble cytochromes and functionally related electron carriers
OOP74025.1	fig|1520.77.peg.2075	CDS	NODE_2_length_829829_cov_83.6585	245838	246209	3	+	372	Ferredoxin	Soluble cytochromes and functionally related electron carriers
OOP73121.1	fig|1520.77.peg.2659	CDS	NODE_3_length_819615_cov_56.6944	2825	2025	-2	-	801	Ferredoxin	Soluble cytochromes and functionally related electron carriers
OOP73270.1	fig|1520.77.peg.2830	CDS	NODE_3_length_819615_cov_56.6944	206144	206905	2	+	762	Ferredoxin	Soluble cytochromes and functionally related electron carriers
OOP73475.1	fig|1520.77.peg.3055	CDS	NODE_3_length_819615_cov_56.6944	443642	442881	-2	-	762	Ferredoxin	Soluble cytochromes and functionally related electron carriers
OOP73056.1	fig|1520.77.peg.3895	CDS	NODE_4_length_604572_cov_120.866	546090	545299	-3	-	792	Ferredoxin	Soluble cytochromes and functionally related electron carriers
OOP72571.1	fig|1520.77.peg.4317	CDS	NODE_5_length_413361_cov_65.9822	376437	376090	-3	-	348	Ferredoxin	Soluble cytochromes and functionally related electron carriers
OOP72237.1	fig|1520.77.peg.4680	CDS	NODE_6_length_383704_cov_86.1472	370360	369602	-1	-	759	Ferredoxin	Soluble cytochromes and functionally related electron carriers

Protein Accession Number	Feature ID (RAST)	Type	Contig	Start	Stop	Frame	Strand	Length (bp)	Function	Subsystem
OOP71734.1	fig|1520.77.peg.4827	CDS	NODE_7_length_339772_cov_75.5879	129714	128941	-3	-	774	Ferredoxin	Soluble cytochromes and functionally related electron carriers
OOP71812.1	fig|1520.77.peg.4836	CDS	NODE_7_length_339772_cov_75.5879	138475	137730	-1	-	744	Ferredoxin	Soluble cytochromes and functionally related electron carriers
OOP71746.1	fig|1520.77.peg.4844	CDS	NODE_7_length_339772_cov_75.5879	144559	145401	1	+	843	Ferredoxin	Soluble cytochromes and functionally related electron carriers
OOP71810.1	fig|1520.77.peg.4914	CDS	NODE_7_length_339772_cov_75.5879	219470	218664	-2	-	807	Ferredoxin	Soluble cytochromes and functionally related electron carriers
OOP71812.1	fig|1520.77.peg.4916	CDS	NODE_7_length_339772_cov_75.5879	221542	220784	-1	-	759	Ferredoxin	Soluble cytochromes and functionally related electron carriers
OOP71417.1	fig|1520.77.peg.5040	CDS	NODE_8_length_237282_cov_113.494	10542	10712	3	+	171	Ferredoxin	Soluble cytochromes and functionally related electron carriers
OOP75134.1	fig|1520.77.peg.940	CDS	NODE_1_length_1193148_cov_98.3687	748817	752326	2	+	3510	Pyruvate-flavodoxin oxidoreductase (EC 1.2.7.-)	Methionine Degradation
OOP74237.1	fig|1520.77.peg.2299	CDS	NODE_2_length_829829_cov_83.6585	480575	477051	-2	-	3525	Pyruvate-flavodoxin oxidoreductase (EC 1.2.7.-)	Methionine Degradation
OOP71310.1	fig|1520.77.peg.5348	CDS	NODE_9_length_221718_cov_69.901	112811	109296	-2	-	3516	Pyruvate-flavodoxin oxidoreductase (EC 1.2.7.-)	Methionine Degradation
OOP71171.1	fig|1520.77.peg.175	CDS	NODE_10_length_219934_cov_78.8972	178923	180308	3	+	1386	Periplasmic [Fe] hydrogenase large subunit (EC 1.12.7.2)	Hydrogenases
OOP75336.1	fig|1520.77.peg.1158	CDS	NODE_1_length_1193148_cov_98.3687	1008324	1010027	3	+	1704	Periplasmic [Fe] hydrogenase large	Hydrogenases

Protein Accession Number	Feature ID (RAST)	Type	Contig	Start	Stop	Frame	Strand	Length (bp)	Function	Subsystem
									subunit (EC 1.12.7.2)	
OOP75440.1	fig|1520.77.peg.1280	CDS	NODE_1_length_1193148_cov_98.3687	1141989	1143569	3	+	1581	Periplasmic [Fe] hydrogenase large subunit (EC 1.12.7.2)	Hydrogenases
OOP71264.1	fig|1520.77.peg.5299	CDS	NODE_9_length_221718_cov_69.901	60512	61861	2	+	1350	Periplasmic [Fe] hydrogenase (EC 1.12.7.2)	Hydrogenases
OOP71388.1	fig|1520.77.peg.5427	CDS	NODE_9_length_221718_cov_69.901	203941	202007	-1	-	1935	Periplasmic [Fe] hydrogenase large subunit (EC 1.12.7.2)	Hydrogenases
OOP72654.1	fig|1520.77.peg.3439	CDS	NODE_4_length_604572_cov_120.866	53067	54248	3	+	1182	Acetyl-CoA acetyltransferase (EC 2.3.1.9)	Acetyl-CoA fermentation to Butyrate
OOP71768.1	fig|1520.77.peg.4868	CDS	NODE_7_length_339772_cov_75.5879	167172	165994	-3	-	1179	Acetyl-CoA acetyltransferase (EC 2.3.1.9)	Acetyl-CoA fermentation to Butyrate
OOP73333.1	fig|1520.77.peg.2901	CDS	NODE_3_length_819615_cov_56.6944	275425	276141	1	+	717	Butyrate-acetoacetate CoA-transferase subunit A (EC 2.8.3.9)	Acetyl-CoA fermentation to Butyrate
OOP73334.1	fig|1520.77.peg.2902	CDS	NODE_3_length_819615_cov_56.6944	276150	276806	3	+	657	Butyrate-acetoacetate CoA-transferase	Acetyl-CoA fermentation to Butyrate

Protein Accession Number	Feature ID (RAST)	Type	Contig	Start	Stop	Frame	Strand	Length (bp)	Function	Subsystem
									subunit B (EC 2.8.3.9)	
OOP72654.1	fig|1520.77.peg.3439	CDS	NODE_4_length_604572_cov_120.866	53067	54248	3	+	1182	Acetyl-CoA acetyltransferase (EC 2.3.1.9)	Acetyl-CoA fermentation to Butyrate
OOP71768.1	fig|1520.77.peg.4868	CDS	NODE_7_length_339772_cov_75.5879	167172	165994	-3	-	1179	Acetyl-CoA acetyltransferase (EC 2.3.1.9)	Acetyl-CoA fermentation to Butyrate
OOP71575.1	fig|1520.77.peg.5202	CDS	NODE_8_length_237282_cov_113.494	183593	184441	2	+	849	3-hydroxybutyryl-CoA dehydrogenase (EC 1.1.1.157)	Acetyl-CoA fermentation to Butyrate
OOP71572.1	fig|1520.77.peg.5199	CDS	NODE_8_length_237282_cov_113.494	180439	181578	1	+	1140	Butyryl-CoA dehydrogenase (EC 1.3.8.1)	5-FCL-like protein
OOP73548.1	fig|1520.77.peg.3127	CDS	NODE_3_length_819615_cov_56.6944	535000	533837	-1	-	1164	NADH-dependent butanol dehydrogenase A (EC 1.1.1.-)	Butanol Biosynthesis
OOP71235.1	fig|1520.77.peg.5267	CDS	NODE_9_length_221718_cov_69.901	25517	24339	-2	-	1179	NADH-dependent butanol dehydrogenase A (EC 1.1.1.-)	Butanol Biosynthesis
OOP75388.1	fig|1520.77.peg.1216	CDS	NODE_1_length_1193148_cov_98.3687	1072491	1073606	3	+	1116	Alcohol dehydrogenase (EC 1.1.1.1)	5-FCL-like protein

Protein Accession Number	Feature ID (RAST)	Type	Contig	Start	Stop	Frame	Strand	Length (bp)	Function	Subsystem
OOP74528.1	fig|1520.77.peg.2621	CDS	NODE_2_length_829829_cov_83.6585	789107	790273	2	+	1167	Alcohol dehydrogenase (EC 1.1.1.1)	Acetaldehyde dehydrogenase (EC 1.2.1.10)
OOP73730.1	fig|1520.77.peg.3326	CDS	NODE_3_length_819615_cov_56.6944	738088	736922	-1	-	1167	Alcohol dehydrogenase (EC 1.1.1.1)	Acetaldehyde dehydrogenase (EC 1.2.1.10)
OOP71652.1	fig|1520.77.peg.4724	CDS	NODE_7_length_339772_cov_75.5879	29199	28177	-3	-	1023	Alcohol dehydrogenase (EC 1.1.1.1)	5-FCL-like protein
OOP71557.1	fig|1520.77.peg.5184	CDS	NODE_8_length_237282_cov_113.494	157628	160222	2	+	2595	Alcohol dehydrogenase (EC 1.1.1.1)	Acetaldehyde dehydrogenase (EC 1.2.1.10)
OOP71098.1	fig|1520.77.peg.93	CDS	NODE_10_length_219934_cov_78.8972	85198	86355	1	+	1158	1,3-propanediol dehydrogenase (EC 1.1.1.202)	- none -
OOP72888.1	fig|1520.77.peg.3696	CDS	NODE_4_length_604572_cov_120.866	321364	322497	1	+	1134	1,3-propanediol dehydrogenase (EC 1.1.1.202)	- none -
